# Ferroptosis-induced SUMO2 lactylation counteracts ferroptosis by enhancing ACSL4 degradation in lung adenocarcinoma

**DOI:** 10.1038/s41421-025-00829-6

**Published:** 2025-10-07

**Authors:** Guangyao Shan, Yunyi Bian, Qihai Sui, Jiaqi Liang, Shencheng Ren, Binyang Pan, Haochun Shi, Zhaolin Zheng, Dejun Zeng, Junkan Zhu, Zhencong Chen, Guoshu Bi, Hong Fan, Cheng Zhan

**Affiliations:** 1 Department of Thoracic Surgery, Zhongshan Hospital, Fudan University, Shanghai, China; 2 Department of Thoracic Surgery, Shanghai Chest Hospital, Shanghai Jiao Tong University School of Medicine, Shanghai, China

**Keywords:** Non-small-cell lung cancer, Cell death

## Abstract

Lactylation, a lactate-mediated post-translational modification, has garnered significant attention for its pivotal role in epigenetic modulation. However, the intricate interplay between lactylation and ferroptosis in lung adenocarcinoma (LUAD) remains to be fully elucidated. Utilizing metabolomic profiling and comprehensive metabolic library screening, our study uncovers that ferroptosis markedly enhances lactic acid accumulation and subsequent protein lactylation, which in turn confers resistance to ferroptosis in LUAD cells. Functional assays, comprising cell viability tests, lipid peroxidation detection, as well as malondialdehyde and glutathione measurements, collectively reveal that SUMO2-K11 lactylation (SUMO2-K11la), the most prominently elevated lactylation in response to ferroptosis induction, serves as a pivotal factor in determining ferroptosis resistance. Sumoylation proteomics and co-immunoprecipitation assays reveal that SUMO2-K11la impairs the interaction between SUMO2 and ACSL4. Consequently, this disruption facilitates the degradation of ACSL4, thereby disrupting lipid metabolism and effectively mitigating ferroptosis. Furthermore, AARS1 is identified as the lactyltransferase and HDAC1 as the delactylase for SUMO2-K11la. Based on these findings, we develop a cell-penetrating peptide that competitively and specifically inhibits SUMO2-K11la. This peptide significantly potentiates ferroptosis and sensitizes LUAD to cisplatin in xenograft models, while enhancing chemoimmunotherapy responses in spontaneous lung cancer models. Overall, our findings imply that SUMO2-K11la is a pivotal regulator of ferroptosis resistance in LUAD, and suggest a promising strategy to potentiate ferroptosis-based cancer therapies via targeting SUMO2-K11la by the cell-penetrating peptide.

## Introduction

As a highly prevalent malignancy, lung cancer is the primary cause of cancer-related mortality globally^1^. Notably, non-small cell lung cancer (NSCLC) accounts for ~85% of all lung cancer cases, with lung adenocarcinoma (LUAD) representing the most frequent subtype^2^. Despite recent advancements in cancer therapy, the prognosis for LUAD patients remains poor, with a five-year survival rate of less than 26%^3^.

Reprogramming of cellular metabolism stands as one of the hallmarks of malignancies^4^. Cancer cells have the capacity to switch their glucose metabolism, and consequently their energy production, predominantly towards glycolysis even under aerobic conditions, which is widely known as “aerobic glycolysis”^5^. The accumulating lactic acid (LA) generated from heightened glycolysis plays a pivotal role in tumor progression, immune response, and drug resistance^6–10^. Recently, beyond its metabolic roles, LA has also been identified as a crucial participant in a novel post-translational modification (PTM) of lysine residue termed lactylation (Kla), thereby regulating transcription via histone modifications and modulating the functions of non-histone proteins^11,12^. However, numerous unresolved questions persist regarding lactylation, especially concerning the lactylation of non-histone proteins within cancer cells, which still warrants further exploration.

Ferroptosis is a regulated cell death characterized by the production of reactive oxygen species (ROS), the accumulation of lipid peroxides, an elevation in intracellular ferrous iron levels, and the inhibition of antioxidant pathways^13,14^. The employment of ferroptosis-inducing strategies has demonstrated remarkable efficacy in overcoming resistance to traditional treatment modalities, including chemotherapy, radiotherapy, and immunotherapy^15^. Accumulating evidence has implicated ferroptosis in numerous cellular metabolic processes, particularly in mitochondrial metabolism, as mitochondria are the primary sites for the generation of ROS. Given that mitochondrial damage is the characteristic of ferroptosis, cells tend to rely more on glycolysis for energy production during ferroptosis, resulting in an increased accumulation of LA^16^. Though previous studies have demonstrated that LA acts as an inhibitor of ferroptosis by modulating lipid metabolism and antioxidant systems^16–18^, the significance of lactylation, another facet of LA’s activity, still needs to be fully elucidated in ferroptosis.

In this investigation, we reported that ferroptosis markedly enhances LA accumulation and subsequent protein lactylation, which in turn confers resistance to ferroptosis in LUAD cells. Moreover, we revealed that SUMO2-K11 lactylation (SUMO2-K11la), a previously unreported and prominently elevated lactylated modification in response to ferroptosis induction, serves as a pivotal factor by inhibiting the sumoylation and facilitating the degradation of ACSL4, thereby mitigating ferroptosis. A cell-penetrating peptide (CPP) was further developed to competitively target SUMO2-K11la, and its ferroptosis-promoting efficacy was rigorously validated in both patient-derived organoids (PDOs) and animal models, including xenograft and spontaneous lung cancer models.

## Results

### Ferroptosis-induced LA production contributes to ferroptosis resistance

**Fig. 1:**
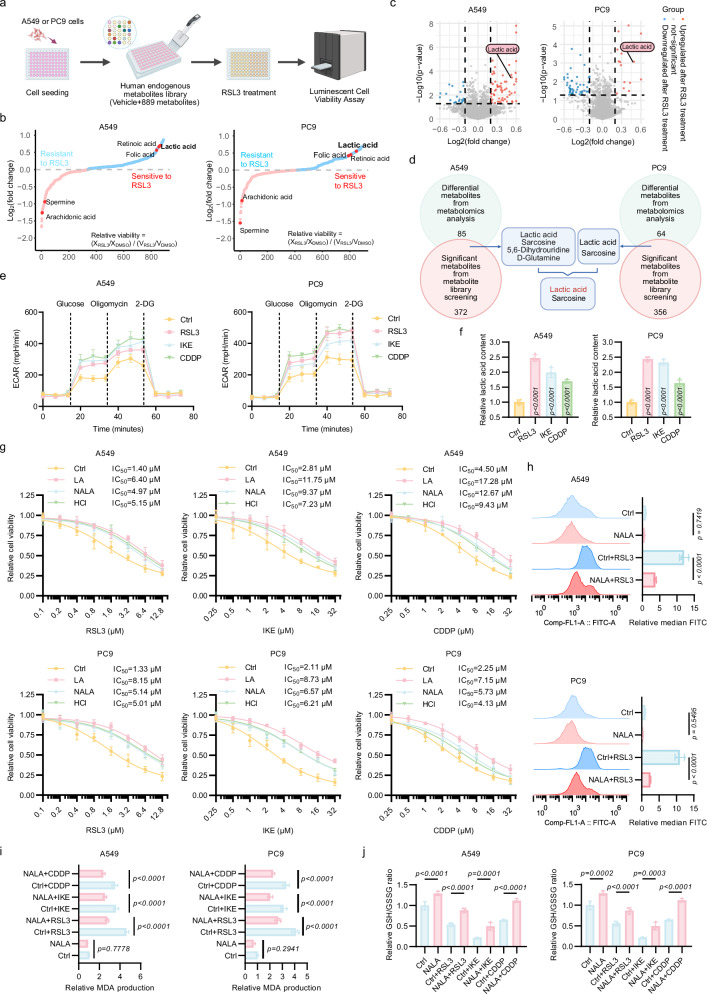
Ferroptosis-induced lactic acid production contributes to ferroptosis resistance. **a** Schematic workflow of human endogenous metabolite library screening in A549 and PC9 cells. **b** Relative viability of lung adenocarcinoma (LUAD) cells pre-treated with indicated metabolites (X) or vehicle control (V), followed by exposure to RSL3 (2 μM) or DMSO for 48 h. **c** Volcano plot of metabolomic profiling in A549 and PC9 cells post-RSL3 treatment (2 μM, 24 h). Metabolites with |log_2_(fold change)| > 0.2 and –log_10_(*p*-value) < 1.301 (dashed lines) were considered statistically significant. **d** Venn diagram highlighting overlapping metabolites identified by dual screening strategies as key regulators of ferroptosis. **e** Extracellular acidification rate (ECAR) in LUAD cells treated with RSL3 (2 μM), IKE (10 μM), or cisplatin (CDDP, 10 μM) for 24 h, measured via Seahorse XF Analyzer. **f** Lactic acid (LA) levels in LUAD cells following 24 h treatment with RSL3 (2 μM), IKE (10 μM), or CDDP (10 μM), quantified using an LA assay kit. **g** Dose-dependent effects of RSL3 (48 h), IKE (72 h), or CDDP (72 h) on A549 cell viability, with or without LA/Sodium lactate (NALA)/HCl supplementation (5 mM) (*n* = 4 biological replicates). **h**–**j** Quantification of ferroptosis-associated biomarkers demonstrated the anti-ferroptotic activity of NALA: lipid-reactive oxygen species (lipid-ROS) were measured via BODIPY-C11 fluorescence probes (**h**), malondialdehyde (MDA) levels were assessed by thiobarbituric acid assay (**i**), and cellular redox status was determined by GSH/GSSG ratio (**j**). Data were analyzed by one-way ANOVA and were presented by mean ± SD.

It is well-established that ferroptosis is intricately intertwined with cellular metabolism, including glucose metabolism, lipid metabolism, and several other metabolic processes^19,20^. To systematically investigate the metabolites implicated in ferroptosis, we integrated a metabolite library screening (|log_2_(relative viability)| > 0.2) with non-targeted metabolomics profiling (|log_2_(fold change)| > 0.2 and -log_10_(*p*-value) < 1.301) (Fig. 1a–c). This dual approach pinpointed LA and sarcosine as key metabolites associated with ferroptotic regulation (Fig. 1d). While sarcosine’s pro-ferroptotic role was previously established by our team^21^, this study focuses on LA, which is upregulated during ferroptosis and exhibits anti-ferroptotic activity.

To delineate ferroptosis-driven metabolic reprogramming, we performed RNA sequencing of PC9 cells treated with ferroptosis inducers (FINs, including RAS-selective lethal 3 (RSL3) or imidazole ketone erastin (IKE)), which showed that the differentially expressed genes were significantly enriched in glycolytic pathways (Supplementary Fig. S1a, b). Given CDDP’s capacity to induce ferroptosis, herein it was also utilized as a FIN as previously reported to enhance the clinical relevance of our study, though it is also relevant to other cell death types^15,22–24^. Subsequently, we performed Seahorse experiments, revealing enhanced glycolysis, and further validated this through LA assay kits, where LA levels increased significantly upon ferroptotic induction (Fig. 1e, f). Since mitochondrial impairment occurs concurrently during ferroptosis and these organelles constitute the predominant sources of ROS, we hypothesized that cells engage compensatory glycolysis as an adaptive metabolic strategy. This metabolic shift maintains ATP production while reducing oxidative stress through suppressed electron transport chain activity, establishing a protective mechanism that balances energy needs and redox homeostasis.

We further interrogated the functional contributions of LA, hydrogen ions (H^+^), and sodium lactate (NALA) to ferroptosis. Cytotoxicity assays revealed that LA, H^+^, and NALA attenuated FIN-induced ferroptotic cell death (Fig. 1g), implicating that both lactate anion and acidic pH contributed to LA’s protective role. As pH-dependent ferroptosis modulation is documented^25,26^, we prioritized investigating lactate anion’s impact. BODIPY-C11 probe-based lipid peroxidation assays confirmed NALA’s capacity to suppress this ferroptotic hallmark (Fig. 1h), corroborated by reduced malondialdehyde (MDA) levels in NALA-treated cells (Fig. 1i). Furthermore, we measured the GSH/GSSG ratio as an indicator of cellular redox status, which showed a significant increase after NALA treatment (Fig. 1j).

### Ferroptosis induces the lactylation of SUMO2 at lysine (K)-11 site

**Fig. 2:**
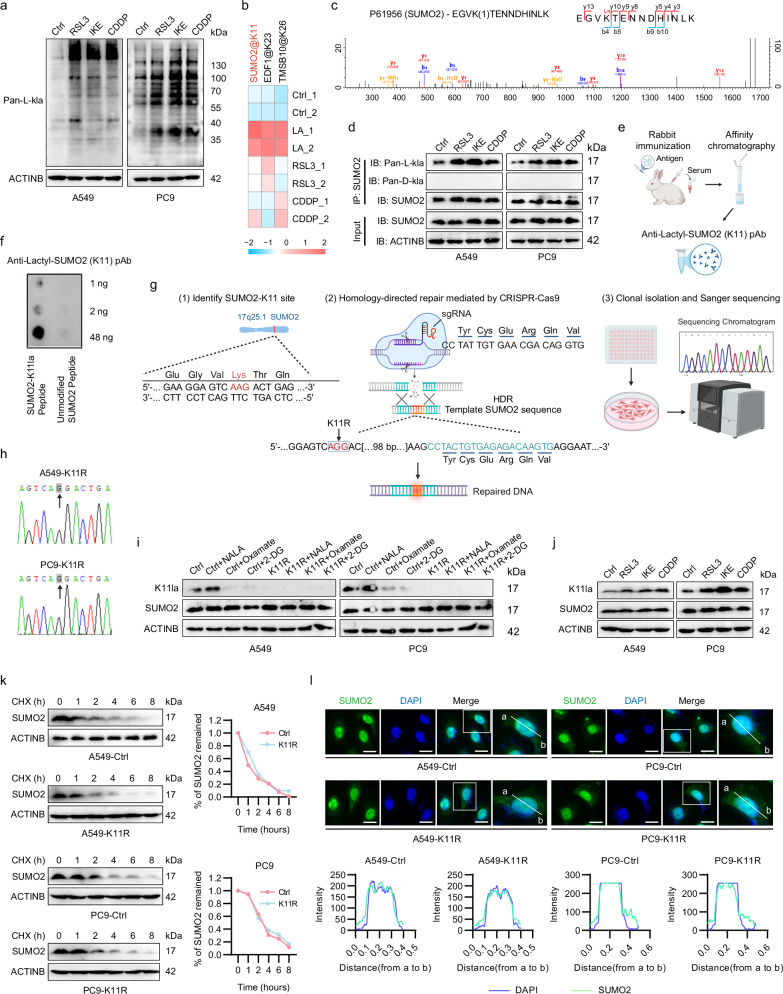
Ferroptosis induces the lactylation of SUMO2 at lysine (K) 11 site. **a** Pan-L-lactylation (Pan-L-Kla) levels were elevated in LUAD cells treated with RSL3 (2 μM), IKE (10 μM), or CDDP (10 μM) for 24 h. **b** Heatmap of three most significant lactylated protein sites identified by mass spectrometry (MS) in A549 cells treated with LA (20 mM), RSL3 (2 μM), or CDDP (10 μM), followed by immunoprecipitation with Pan-L-Kla antibody or IgG. **c** The B-y ion matching diagram of the lactylated SUMO2-K11 site (SUMO2-K11la). **d** Co-immunoprecipitation (Co-IP) assays showing enhanced SUMO2 lactylation level upon RSL3 (2 μM), IKE (10 μM), or CDDP (10 μM) treatment for 24 h. **e** Schematic diagram of customized SUMO2-K11la antibody development. **f** Dot blot validating the specificity for SUMO2-K11la antibody. **g** CRISPR-Cas9/homology-directed repair (HDR)-mediated generation of lactylation-deficient (Lysine 11 to Arginine, K11R) LUAD cells. **h** Sanger sequencing confirming the introduction of K11R mutations in A549 and PC9 cells. **i** SUMO2-K11la levels modulated by NALA (5 mM), sodium oxamate (5 mM), or 2-DG (5 mM) in Control (Ctrl) and K11R cells. **j** The SUMO2-K11la level was raised upon RSL3 (2 μM), IKE (10 μM), or CDDP (10 μM) treatment for 24 h. **k** Cycloheximide (CHX) chase assays showing unaltered SUMO2 protein stability in K11R mutants. **l** Immunofluorescence of SUMO2 (green) and DAPI (blue) confirming unchanged subcellular localization in K11R mutants. Scale bars, 20 μm. Data were presented by mean ± SD.

Considering that lactate anion is highly implicated in lactylation^27–29^, herein we delved into the connection between lactylation and ferroptosis. After treating LUAD cells with RSL3, IKE, and CDDP, we found a significant increase in global protein lactylation levels upon ferroptosis induction using a pan-L-kla antibody (Fig. 2a). To identify specific Kla sites, mass spectrometry analyses were performed after enrichment using pan-L-kla antibody-coupled beads (Supplementary Data S1). Our results demonstrated that SUMO2, which is responsible for another PTM called sumoylation, exhibited the most pronounced increase in lactylated intensity at the K11 site upon ferroptosis induction (Fig. 2b, c; Supplementary Fig. S2a).

To ensure the accuracy of the lactylation at the SUMO2-K11 site, we examined previously published lactylomics data and found that SUMO2-K11la was also detected in previous studies involving NSCLC and hepatocellular carcinoma, but has not been explored^30,31^ (Supplementary Fig. S2b). Notably, K11 is highly conserved in SUMO2 across various species, suggesting that this site may play a crucial role in SUMO2 function (Supplementary Fig. S2c). Additionally, gene set enrichment analysis conducted on LUAD patients from the TCGA dataset revealed a significant correlation between SUMO2 expression and cellular response to stress (Supplementary Fig. S2d), indicating that SUMO2 plays an important role in the cellular response to stresses such as ferroptosis. Co-immunoprecipitation (Co-IP) analysis confirmed that SUMO2 lysine L-lactylation, instead of D-lactylation levels, increased under ferroptotic induction^32^, while the SUMO2 mRNA and protein levels remained unchanged (Fig. 2d; Supplementary Fig. S2e).

Subsequently, we customized an anti-Lactyl-SUMO2 (K11) poly-antibody and confirmed its specificity for lactylated SUMO2-K11 peptide (Fig. 2e, f; Supplementary Fig. S2f). To specifically investigate the consequences of SUMO2-K11la, we exploited CRISPR/Cas9-mediated gene editing to generate K11R-mutant cell lines (Fig. 2g, h), a mutant form where the lysine at the 11th site was replaced with arginine (R), rendering it unable to undergo lactylation. Treatment of LUAD cells with NALA increased SUMO2-K11la levels, while sodium oxamate, an LDH inhibitor, and 2-Deoxy-D-glucose (2-DG), a glycolysis inhibitor, decreased SUMO2-K11la levels (Fig. 2i). Besides, the SUMO2-K11la was undetectable in K11R cells, demonstrating the specificity of the antibody (Fig. 2i). The Co-IP assays coupled with pan-L-kla antibody demonstrated a dose-dependent increase in both global lactylation and K11-site-specific lactylation upon NALA treatment (Supplementary Fig. S2g). Consistent with the aforementioned findings, FIN treatment also significantly elevated SUMO2-K11la levels (Fig. 2j).

Next, we assessed the impact of SUMO2-K11la on the function of SUMO2 through protein half-life analysis and immunofluorescence staining. Cycloheximide (CHX) chase assays demonstrated that SUMO2-K11la did not affect SUMO2 protein stability (Fig. 2k). Additionally, no significant changes were observed in SUMO2 subcellular distribution upon K11 lactylation (Fig. 2l).

### SUMO2-K11la counteracts ferroptosis in LUAD

**Fig. 3:**
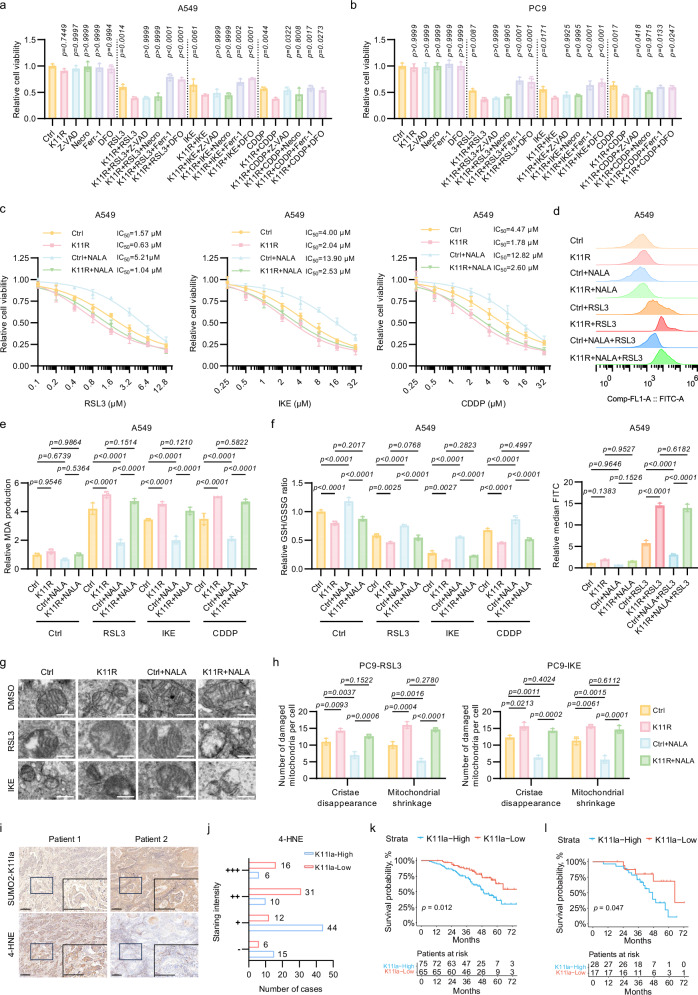
SUMO2-K11la counteracts ferroptosis in LUAD. **a**, **b** Ferroptosis inhibitors ferrostatin-1 (Ferr-1, 10 μM) and deferoxamine (DFO, 10 μM) reversed K11R-driven cell death in LUAD cells, while apoptosis inhibitor Z-VAD-FMK (10 μM) and necroptosis inhibitor Necrosulfonamide (Necro, 10 μM) had no significant effect. **c** Relative viability of A549 Ctrl and K11R cells following treatment with varying concentrations of RSL3 (48 h), IKE (72 h), or CDDP (72 h) with or without concurrent incubation with NALA (5 mM). **d**–**f** Quantification of ferroptosis-associated biomarkers demonstrated the ferroptosis-promoting effect of K11R mutation: lipid-ROS were measured via BODIPY-C11 fluorescence probes (**d**), MDA levels were assessed by thiobarbituric acid assay (**e**), and cellular redox status was determined by GSH/GSSG ratio (**f**). **g**, **h** Transmission electron microscopy revealed pronounced mitochondrial damage (cristae disappearance and mitochondrial shrinkage) in K11R cells, which is not significantly affected by NALA supplementation. Scale bars, 5 μm. **i**, **j** Immunohistochemistry of LUAD patient tissues demonstrated an inverse correlation between SUMO2-K11la levels and lipid peroxidation marker 4-hydroxynonenal (4-HNE). Scale bars, 200 μm. **k**, **l** High SUMO2-K11la expression predicted reduced overall survival in 140 LUAD patients (**k**) and 45 CDDP-treated patients (**l**). Statistical analysis was performed using one-way ANOVA, and results were presented as mean ± SD.

To test the effect of SUMO2-K11la on ferroptosis, cytotoxicity assays were performed, and the results demonstrated that the introduction of K11R mutation exhibited heightened sensitivity to ferroptosis compared to the control (Ctrl) group (Fig. 3a, b). Notably, treatment with apoptosis inhibitors (Z-VAD-FMK, Z-VAD) or necroptosis inhibitors (Necrosulfonamide, Necro) failed to rescue K11R-induced cell death, whereas ferroptosis-specific inhibitors (deferoxamine (DFO) and ferrostatin-1 (Ferr-1)) elicited a marked rescue effect, confirming ferroptosis as the primary death mechanism (Fig. 3a, b). IC_50_ quantification further demonstrated that K11R significantly sensitized cells to FINs, while NALA conferred resistance in Ctrl cells but had negligible effects in K11R mutants (Fig. 3c; Supplementary Fig. S3a).

As shown in Fig. 3d and Supplementary Fig. S3b, lipid peroxidation, the key characteristic of ferroptosis, was significantly elevated in the K11R mutant group compared to the Ctrl group. NALA suppressed peroxidation in Ctrl cells but failed to mitigate it in mutants. MDA detection and GSH/GSSG measurement also supported the pro-ferroptosis role of K11R mutation (Fig. 3e, f; Supplementary Fig. S3c, d). Moreover, our electron microscopy showed that mitochondrial damage, another typical feature during ferroptosis, was more pronounced in the K11R mutant group compared to the Ctrl group, including membrane disruption and loss of cristae structure (Fig. 3g, h). Collectively, these results provided compelling evidence that the SUMO2-K11la counteracts ferroptosis in LUAD.

To elucidate the clinical relevance of SUMO2-K11la level, we conducted immunohistochemical (IHC) analysis of tumor specimens from 140 LUAD patients. The specificity of the SUMO2-K11a antibody was rigorously confirmed through peptide competition assays. Quantitative IHC scoring demonstrated that SUMO2-K11la levels could lead to diminished ferroptosis activity, as evidenced by reduced 4-Hydroxynonenal (4-HNE) staining intensity (Fig. 3i, j). Kaplan–Meier survival analysis revealed that high SUMO2-K11la level was associated with poor prognosis in LUAD patients as well as those who received postoperative chemotherapy (Fig. 3k, l; Supplementary Tables S1, S2). These findings suggest that SUMO2-K11la level could serve as a novel prognostic biomarker in LUAD.

### SUMO2-K11la hinders the sumoylation of ACSL4 at K500 site to promote ACSL4 degradation

**Fig. 4:**
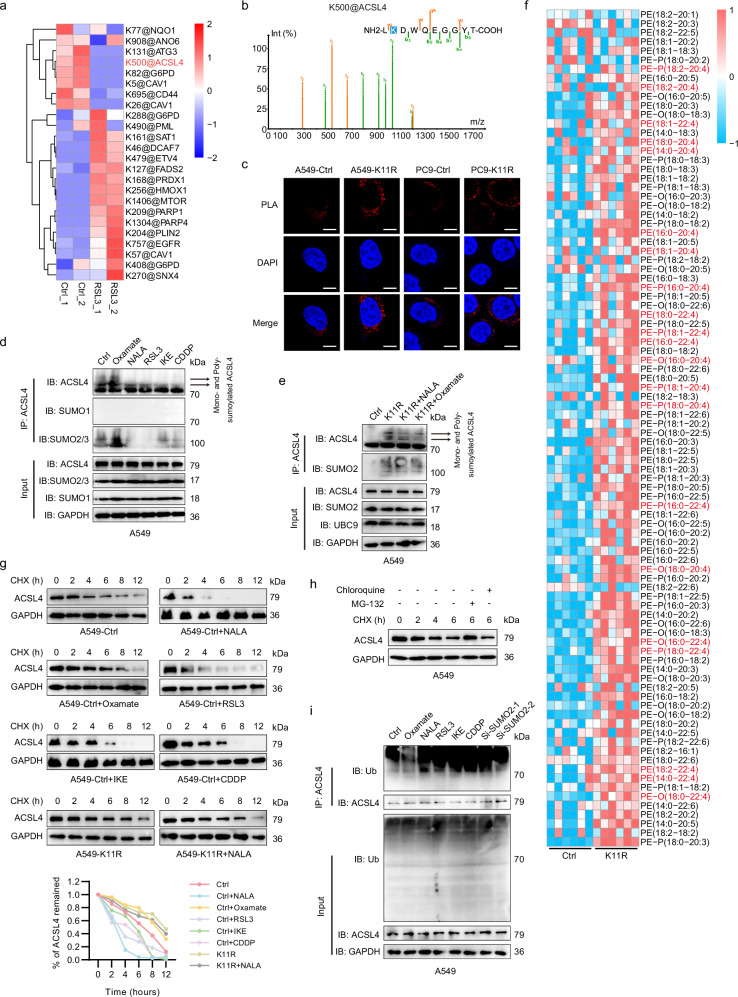
SUMO2-K11la hinders the sumoylation of ACSL4 at K500 site to promote ACSL4 degradation. **a** Quantitative SUMOylome profiling in LUAD cells under RSL3-induced ferroptosis (2 μM, 24 h) identified ACSL4-K500 as a dynamically regulated sumoylation site. **b** The B-y ion matching diagram of ACSL4-K500 sumoylated site. **c** Proximity ligation assay (PLA) confirmed enhanced SUMO2-ACSL4 interaction in SUMO2-K11R mutants. PLA: red; DAPI: blue. Scale bars, 10 μm. **d** Co-IP assays demonstrated suppression of SUMO2/3-mediated ACSL4 sumoylation by NALA or FINs, and its augmentation by sodium oxamate. **e** SUMO2-K11R mutation abolished lactylation-dependent regulation and hyper-sumoylated ACSL4. **f** Lipidomics revealed elevated PE-arachidonic acid (PE-AdA, 20:4) and PE-adrenic acid (PE-AA, 22:4) in SUMO2-K11R cells. **g** CHX chase assays showed accelerated ACSL4 degradation upon NALA/FIN treatment, reversed by sodium oxamate or SUMO2-K11R in A549 cells. **h** Proteasomal inhibitor MG132 (10 μM), but not lysosomal inhibitor chloroquine (20 μM), rescued ACSL4 degradation in A549 cells. **i** Ubiquitination assays indicated increased polyubiquitination of ACSL4 following NALA/FIN treatment or SUMO2 knockdown, contrasting with sodium oxamate-mediated suppression in A549 cells.

Since SUMO2 is a major contributor of sumoylation, the effect of K11la on the sumoylome in LUAD cells was investigated. Utilizing a 4D label-free quantitative proteomics approach to map SUMOylation dynamics following RSL3-induced ferroptosis, we identified 217 upregulated SUMOylation sites (fold change > 1.2) across 191 proteins and 152 downregulated sites (fold change < 0.8) in 131 proteins (Supplementary Data S2). Notably, we observed that the sumoylation level at the lysine 500 site of ACSL4, a key driver of ferroptosis, was abundant in control cells but virtually absent post-RSL3 treatment (Fig. 4a, b). Consistently, the sumoylation site prediction website GPS-SUMO also corroborated K500 as a sumoylation site of ACSL4 (Supplementary Fig. S4a). Confocal microscopy-based immunofluorescence staining demonstrated spatial co-localization of SUMO2 and ACSL4 in LUAD cells, which remained unaltered by either SUMO2-K11R mutation or FIN treatment (Supplementary Fig. S4b). Proximity ligation assay (PLA) further validated direct physical interaction between SUMO2 and ACSL4, with the SUMO2-K11R mutation markedly enhancing this interaction (Fig. 4c). These findings indicated that while FINs do not alter the subcellular distribution of SUMO2 or ACSL4, they modulate their interaction dynamics. Co-IP analysis (MG-132 was applied to block protein degradation) revealed that NALA or FINs suppressed mono- and poly-SUMO2/3 (but not SUMO1)-mediated ACSL4 SUMOylation, whereas lactate dehydrogenase inhibition (sodium oxamate) elevated it (Fig. 4d; Supplementary Fig. S4c). Critically, the SUMO2-K11R mutation abolished these regulatory effects and hyper-SUMOylated ACSL4, implicating K11la as a negative modulator of SUMO2-ACSL4 interaction (Fig. 4e; Supplementary Fig. S4d).

Given ACSL4 involvement in the conversion of PUFA to PUFA-CoA, we conducted a lipidomics analysis to assess the impact of the K11R mutation on lipid metabolism, which revealed an elevation of phosphatidylethanolamine-arachidonic acid (PE-AdA) and PE-adrenic acid (AA) levels in K11R cells (Fig. 4f). CHX chase assays revealed accelerated degradation of ACSL4 upon NALA/FIN treatment, whereas sodium oxamate or the K11R mutation exerted the opposite effect (Fig. 4g; Supplementary Fig. S4e). Notably, this protein degradation could be rescued by the proteasome inhibitor MG132, instead of the lysosomal inhibitor chloroquine (Fig. 4h; Supplementary Fig. S4f). Ubiquitination assays confirmed that NALA/FIN treatment or SUMO2 knockdown promoted ACSL4 ubiquitination, whereas sodium oxamate reduced it (Fig. 4i; Supplementary Fig. S4g–i). Taken together, our findings suggested that SUMO2-K11la leads to ACSL4 instability by inhibiting its sumoylation and enhancing ubiquitin-mediated proteasomal degradation.

**Fig. 5:**
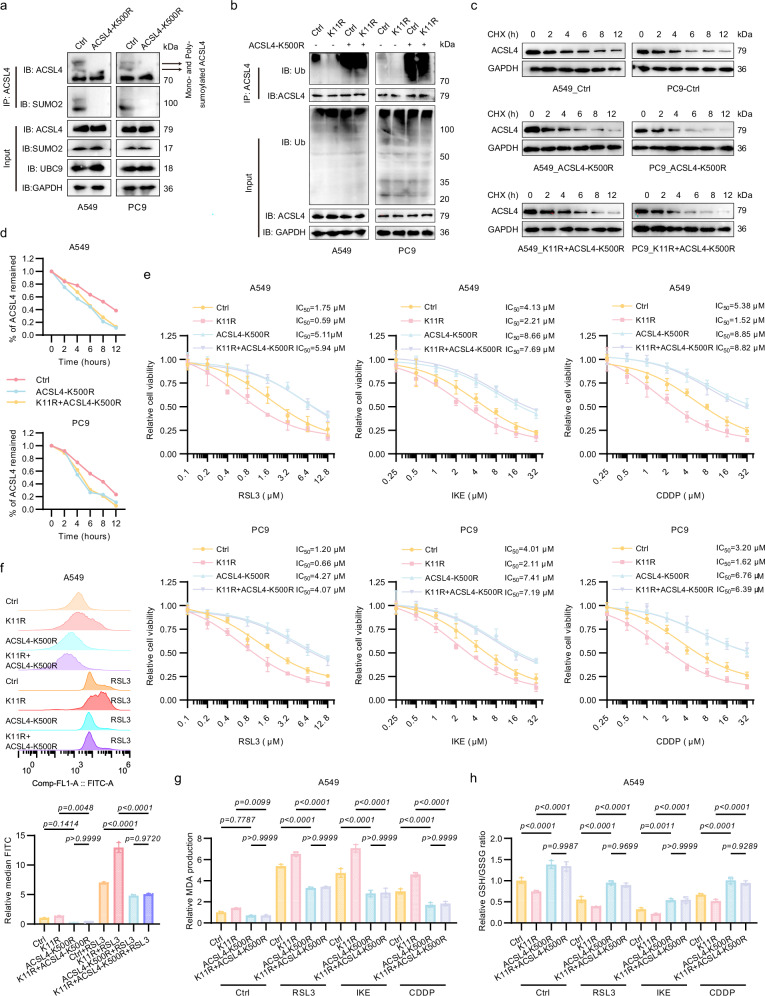
ACSL4-K500R mutation abrogates the effect of SUMO2-K11la on ferroptosis. **a** ACSL4-K500R mutation abolished SUMOylation in LUAD cells. **b**–**d** K500R accelerated ubiquitination-dependent ACSL4 degradation and nullified SUMO2-K11R-induced stabilization in A549 cells. **e**–**h** K500R mutants conferred ferroptosis resistance and abolished the ferroptosis-promoting effect of K11R, as evidenced by cytotoxicity assays (**e**), lipid peroxidation detection (**f**), MDA (**g**), and GSH/GSSG measurements (**h**). Data were analyzed by one-way ANOVA and were presented by mean ± SD.

To directly interrogate ACSL4-K500 SUMOylation in this axis, SUMOylation-deficient ACSL4-K500R LUAD cells were generated (Supplementary Fig. S5a, b). Furthermore, dual-mutant SUMO2-K11R/ACSL4-K500R LUAD cells were generated via CRISPR Cas9-mediated introduction of the ACSL4-K500R mutation into SUMO2-K11R backgrounds. K500R abolished ACSL4 SUMOylation (Fig. 5a), accelerated its ubiquitination-dependent degradation, and nullified the stabilizing effect of SUMO2-K11R (Fig. 5b–d; Supplementary Fig. S5c). Dual mutation of SUMO2-K11R and ACSL4-K500R abrogated the ferroptosis resistance conferred by SUMO2-K11R alone, as evidenced by restored cytotoxicity (Fig. 5e), lipid peroxidation (Fig. 5f), and redox imbalance (Fig. 5g, h; Supplementary Fig. S5d, e).

### AARS1 and HDAC1 are the lactyltransferase and delactylase for SUMO2-K11la, respectively

**Fig. 6:**
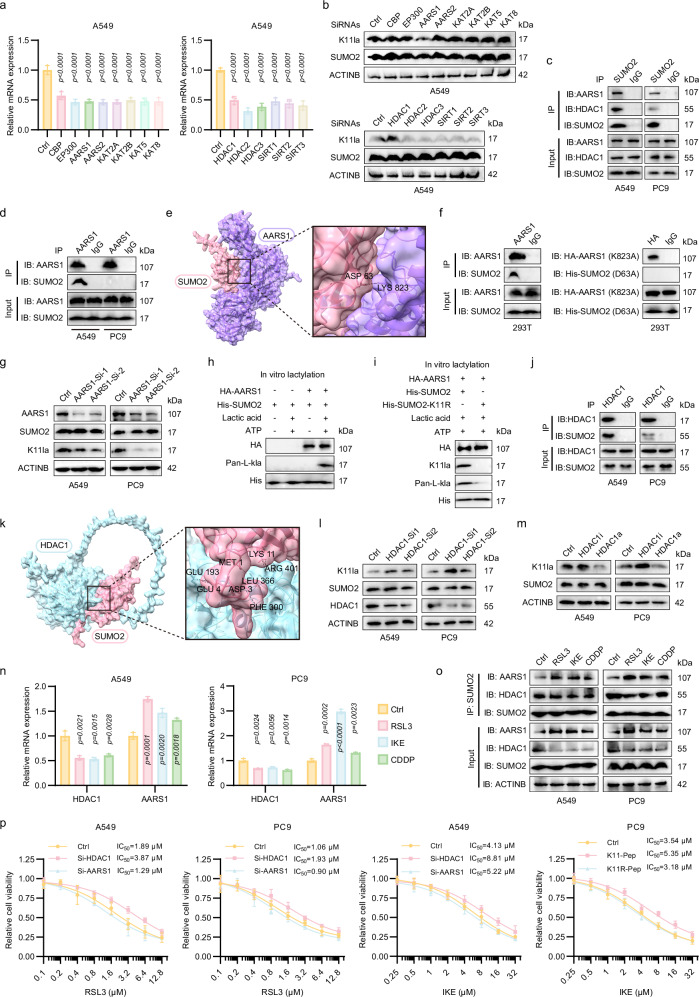
AARS1 and HDAC1 are the lactyltransferase and delactylase for SUMO2-K11la, respectively. **a**, **b** siRNA screening identified AARS1 and HDAC1 as regulators of SUMO2-K11la. **c** Co-IP confirmed SUMO2 interaction with AARS1 and HDAC1. **d** Co-IP assays using AARS1 antibody validated the SUMO2-AARS1 interaction. **e** AlphaFold3-predicted binding interface between AARS1 (Lys823) and SUMO2 (Asp63). **f** Mutagenesis (AARS1-K823A/SUMO2-D63A) abolished SUMO2-AARS1 interaction in 293T cells. **g** AARS1 silencing suppressed K11la in LUAD cells. **h**, **i** In-vitro lactylation assays confirmed that AARS1 is the lactyltransferase of SUMO2-K11 site. **j**, **k** Co-IP assays using HDAC1 antibody and molecular docking validated SUMO2-HDAC1 interaction. **l**, **m** HDAC1 inhibition (quisinostat 2HCl) or knockdown elevates K11la, while activation (exifone) suppresses it, defining HDAC1 as the “eraser.” **n**, **o** FINs upregulated AARS1 and downregulated HDAC1, driving SUMO2-AARS1 binding and weakening SUMO2-HDAC1 interaction. **p** Relative viability of Ctrl, AARS1-knockdown, and HDAC1-knockdown cells following treatment with varying concentrations of RSL3 (48 h) or IKE (72 h). Data were analyzed by one-way ANOVA and were presented by mean ± SD.

To delineate the enzymatic regulation of SUMO2-K11la, we conducted a siRNA-mediated silencing screen targeting eight putative lactyltransferases and six delactylases (Fig. 6a)^31,33–35^. Among these, only AARS1 knockdown significantly reduced SUMO2-K11la levels, whereas HDAC1 depletion elevated this modification, implicating AARS1 as a potential lactyltransferase and HDAC1 as a delactylase (Fig. 6b). Co-IP assays confirmed interactions between SUMO2 and AARS1/HDAC1 (Fig. 6c).

For AARS1, Co-IP in LUAD cells validated its binding to SUMO2 (Fig. 6d). Molecular docking simulations using AlphaFold3 predicted a high-affinity interaction between AARS1 (Lys 823) and SUMO2 (Asp 63)^36^ (Fig. 6e). Mutagenesis of these residues (AARS1-K823A and SUMO2-D63A) abolished binding, confirming their functional necessity (Fig. 6f). siRNA-mediated AARS1 silencing in LUAD cells markedly reduced SUMO2-K11la levels (Fig. 6g). Furthermore, in vitro lactylation assays demonstrated that recombinant AARS1 catalyzes lactate transfer to the SUMO2-K11 site in an ATP- and LA-dependent manner, establishing AARS1 as the enzymatic “writer” of this modification (Fig. 6h, i).

For HDAC1, Co-IP and computational docking confirmed its interaction with SUMO2 (Fig. 6j, k). Pharmacological inhibition or genetic ablation of HDAC1 increased SUMO2-K11la levels, while HDAC1 activation suppressed this modification, identifying HDAC1 as the “eraser” enzyme (Fig. 6l, m).

Ferroptosis stress triggered coupled transcriptional reprogramming in LUAD cells, upregulating AARS1 and downregulating HDAC1 at mRNA and protein levels (Fig. 6n, o). Concomitantly, FINs promoted SUMO2-AARS1 binding while attenuating SUMO2-HDAC1 interactions, suggesting a feedback mechanism linking ferroptotic stress to lactylation dynamics (Fig. 6o). Cytotoxicity assays revealed that AARS1 conferred modest cytoprotection against RSL3/IKE-induced ferroptosis, whereas HDAC1 potentiated ferroptosis sensitivity (Fig. 6p).

### Targeting SUMO2-K11la via a peptidic inhibitor sensitizes LUAD to ferroptosis and chemotherapy plus immunotherapy

**Fig. 7:**
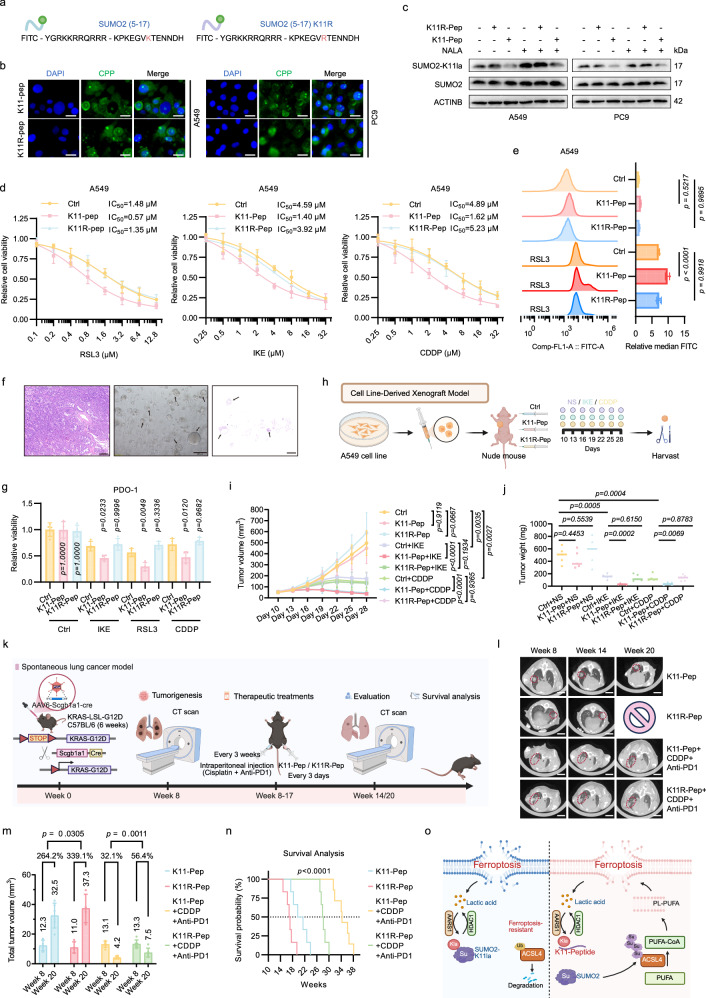
Targeting SUMO2-K11la via a peptidic inhibitor sensitizes LUAD to ferroptosis and chemotherapy plus immunotherapy. **a** Design of FITC-cell penetrating peptide (CPP)-conjugated SUMO2-K11 peptide (K11-Pep) and negative control (K11R-Pep). **b** Fluorescence imaging confirmed nuclear/cytoplasmic peptide delivery (Peptide: Green; DAPI: blue). Scale bars, 20 μm. **c** K11-Pep selectively inhibited SUMO2-K11la levels in LUAD cells. **d** Dose-dependent sensitization of LUAD cells to RSL3, IKE, and CDDP by K11-Pep, quantified via viability assays. A549 cells were treated with indicated concentrations of RSL3 (48 h), IKE (72 h), or CDDP (72 h), with or without co-incubation with indicated CPPs (10 μM). **e** Lipid-ROS measurement demonstrated K11-Pep-driven ferroptosis potentiation. A549 cells were pre-incubated with CPP (10 μM) for 24 h, followed by treatment of RSL3 (2 μM) for 8 h. **f** The representative H&E staining image of the resected LUAD tumor tissue (left), brightfield image of corresponding patient-derived organoid (PDO)-1 (middle), and H&E staining image of PDO-1 (right). Scale bars, 200 μm. **g** K11-Pep sensitized PDOs to ferroptosis and chemotherapy. PDOs were treated with IKE (20 μM), RSL3 (10 μM), or CDDP (30 μM) for 120 h, with or without co-incubation of K11-Pep (10 μM) or K11R-Pep (10 μM). **h** Schematic representation of study design in nude mice (*n* = 5 for each group). **i**, **j** The tumor growth curve and weight of the different groups. **k** Construction of spontaneous lung cancer model in C57BL/6 mice (*n* = 6 for each group). **l**, **m** K11-Pep treatment significantly reduced tumor burden in chemoimmunotherapy-treated C57BL/6 mice. Tumor burden (total tumor volume) was calculated using the formula (length × width²)/2 across all CT scan layers. Lesions greater than 2 mm at week 8 were documented. Scale bars, 5 mm. **n** Survival curves demonstrated the therapeutic benefit of K11-Pep treatment. **o** Model for SUMO2-K11la inhibiting ferroptosis in LUAD. Data were analyzed by one-way or two-way ANOVA and were presented by mean ± SD.

Given the pleiotropic effects of targeting AARS1 or LA biosynthesis, we sought to devise a strategy to selectively perturb SUMO2-K11la. Inspired by prior reports on peptide-based competitive inhibition of PTMs^37–39^, we designed a CPP-conjugated construct, K11-Pep (sequence: KPKEGVKTENNDH), to target the SUMO2 K11 site, alongside a negative control peptide (K11R-Pep, sequence: KPKEGVRTENNDH) based on the sequence surrounding the SUMO2 K11 site. Both peptides were fused to the YGRKKRRQRRR CPP domain, a well-established vehicle for intracellular cargo delivery (Fig. 7a)^40,41^. Next, we confirmed that these peptides exhibited high membrane penetration capability and could effectively enter nuclei and cytoplasm independent of any transfection reagents (Fig. 7b). Western blot analysis revealed that K11-Pep, instead of K11R-Pep, significantly inhibited SUMO2-K11la (Fig. 7c). Intriguingly, K11-Pep augmented ACSL4 sumoylation and attenuated its degradation, implicating that K11-Pep could modulate ACSL4 stability (Supplementary Fig. S6a–c). Functional assays further demonstrated that K11-Pep sensitized cells to ferroptosis, as evidenced by elevated cytotoxicity and lipid peroxidation (Fig. 7d, e; Supplementary Fig. S6d, e).

PDOs demonstrated a notable capability to recapitulate the histological and molecular features of their respective primary tissues, thereby establishing themselves as an optimal model for drug sensitivity assessments^42^. Brightfield and hematoxylin and eosin (H&E) staining confirmed the successful construction of LUAD organoids (Fig. 7f; Supplementary Fig. S6f). The 3D cell viability assays revealed that K11-Pep significantly sensitized PDOs to ferroptosis and CDDP compared to the control group (Fig. 7g; Supplementary Fig. S6g, h). Cell-derived xenograft model was utilized to assess the therapeutic potential of K11-Pep in vivo, and here IKE was used due to its superior metabolic stability, bioavailability, and water solubility in animal models (Fig. 7h)^43^. The results demonstrated that K11-Pep, instead of K11R-Pep, conferred sensitivity to IKE as well as CDDP and significantly inhibited tumor growth (Fig. 7i, j; Supplementary Fig. S7a). More importantly, the administration did not cause significant changes in the hematological parameters and major organs, including the heart, liver, spleen, lung, and kidney, confirming the safety of the CPP treatment (Supplementary Fig. S7b, c).

Single-cell RNA sequencing (scRNA-seq) data from LUAD patients revealed that SUMO2 is predominantly expressed in tumor cells, indicating the specificity of the K11-Pep peptide (Supplementary Fig. S8a, b). Multiplex IHC staining of patient samples further demonstrated minimal co-localization of CD8/CD4 and SUMO2-K11la, indicating that K11-Pep is less likely to exert an impact on T cells (Supplementary Fig. S8c). To assess the potential therapeutic efficacy of K11-Pep in chemoimmunotherapy, a spontaneous lung cancer model was employed (Fig. 7k). Computed tomography (CT) scanning conducted before and after treatment with CDDP plus anti-PD1 antibody demonstrated a significant reduction in tumor burden in the K11-Pep group (Fig. 7l, m). Likewise, survival analysis indicated a significant extension of survival in the K11-Pep group compared to the K11R-Pep group receiving chemoimmunotherapy (Fig. 7n). Flow cytometry analysis confirmed that K11-Pep treatment increased the proportions of CD8^+^ and CD4^+^ T cells within the tumor microenvironment (Supplementary Fig. S8d, e). Elevated levels of lipid peroxidation markers (MDA and 4-HNE) in K11-Pep-treated mice implicated ferroptosis induction as a mechanistic contributor to therapeutic efficacy (Supplementary Fig. S8f, g). These data align with reported links between ferroptotic tumor cell death and potentiated T cell activity^44–47^, proposing a model wherein K11-Pep augments chemoimmunotherapy outcomes through coordinated ferroptosis sensitization and immunomodulation.

These results collectively demonstrated the therapeutic potential of K11-Pep in inhibiting SUMO2-K11la, promoting ferroptosis, and enhancing the efficacy of chemotherapy and chemoimmunotherapy in lung cancer models.

## Discussion

Ferroptosis, a lipid peroxidation-driven form of regulated cell death, represents a promising avenue to overcome therapeutic resistance across multiple treatment modalities, including chemotherapy and immunotherapy^48^. Here, we reported a novel regulatory mechanism wherein SUMO2-K11la emerges as a critical suppressor of ferroptosis through its disruption of ACSL4-SUMO2 interactions, thereby promoting ubiquitination-dependent ACSL4 proteolysis. This discovery is further leveraged to develop a K11-specific CPP that effectively neutralizes SUMO2-K11la activity, which potentiates ferroptosis and enhances susceptibility to chemoimmunotherapy (Fig. 7o).

It is universally recognized that LA is not only a metabolic byproduct but a key player in tumor progression and drug resistance. Previous studies have shown that LA can suppress ferroptosis through activation of the AMPK/SCD1 pathway^17^. Chen et al. also reported elevated LA levels in CDDP-resistant cells, with LDHA expression serving as a negative prognostic indicator in gastric cancer patients. Besides, LA was found to suppress the anti-tumor immunity of T cells and immunotherapy response^49,50^. Beyond its metabolic function, LA also exerts non-metabolic effects via lactylation modifications^51–56^. For example, lactylation at the K247 residue of XRCC1 promotes its nuclear translocation and DNA repair capabilities in glioma^57^. Wang et al. reported that hyperlactylated YY1 directly enhances FGF2 transcription in microglia and promotes angiogenesis^58^. A recent study by Zhang et al. ^59^ demonstrated increased levels of lactylation modifications in NSCLC tissues, which correlated positively with tumor stage and T classification, suggesting a link between elevated lactylation and poor prognosis. In this study, we report a previously unrecognized metabolic reprogramming event wherein ferroptosis induction triggers an enhancement of glycolysis and LA production, culminating in elevated cellular lactylation. Strikingly, this metabolic shift augmented SUMO2 lactylation at the K11 residue during ferroptosis execution. Likewise, we analyzed previously reported lactylome studies, which revealed a conserved pattern of SUMO2-K11 lactylation in NSCLC and hepatocellular carcinoma^30,31^. Our work established, for the first time, a theoretical framework linking SUMO2 lactylation to ferroptosis regulation, offering a paradigm-shifting strategy to promote chemosensitivity through targeted manipulation of this previously unrecognized signaling axis.

Intriguingly, SUMO2 is a major contributor of sumoylation, a process in which SUMO conjugates to the lysine residue of substrate proteins through an enzymatic cascade. This PTM is conserved across all eukaryotes and is crucial in maintaining genomic integrity and modulating intracellular signaling^60^. Aberrant activation of sumoylation has been implicated in various cancers, contributing to tumorigenesis, epithelial-mesenchymal transition, metastasis, and drug resistance^61^. For instance, loss of sumoylation on the tumor suppressor RB1 enhances its transcriptional repression of E2F, thus regulating cell cycle progression^62^. Notably, distinct modification patterns and functions have been observed for SUMO1 and SUMO2/3. SUMO1 typically modifies a diverse range of proteins in a monomeric form and is mainly involved in processes such as transcriptional regulation and nuclear transport, whereas SUMO2/3 usually modifies stress-responsive proteins in either monomeric or polymeric forms, forming polySUMO chains to participate in cellular stress responses and protein degradation^61,63,64^. Stressors such as heat, osmotic shock, and ROS can upregulate cellular sumoylation, primarily through the chain-forming SUMO2/3 paralogues, while compromised sumoylation sensitizes cells to stress, highlighting its role as a crucial anti-stress regulator^65,66^. It is also reported that sumoylation enhances the ROS scavenging activity of Nrf2 to render hepatocellular carcinoma cells more resistant to oxidative stress^67^. Furthermore, consistent with previously reported lactylome studies, our findings revealed that the K11 lactylation does not exist in SUMO3, despite it sharing 96% homology with its paralogue SUMO2, which perhaps contributes to the differences between the functions of SUMO2 and SUMO3.

Combining high-throughput SUMOylome profiling with mechanistic validation, we revealed that ACSL4, a key enzyme regulating PUFA metabolism, underwent SUMO2-mediated desumoylation through both monomeric and polymeric conjugation at the K500 site under ferroptotic stress. Prior studies indicated that lactylation may induce steric hindrance, conformational rearrangements, or charge neutralization in substrate proteins^12^. Building on this, we hypothesized that K11la in SUMO2 disrupts its molecular recognition by ACSL4 and/or SUMOylation-associated enzymes, potentially impeding SUMO2-mediated interactions. Furthermore, as K11-linked poly-SUMO2 chains constitute the predominant polymeric configuration^61,68^, lactylation at this residue likely impedes chain elongation, thereby compromising multivalent interaction. The ACSL4-K500R mutant displayed accelerated degradation compared to wild-type controls and abolished SUMO2-K11R-mediated suppression of ferroptosis. Intriguingly, ubiquitin proteomics in NSCLC cells previously identified K498 and K702, but not K500, as primary ubiquitination sites on ACSL4^69^. Meanwhile, our ubiquitination Co-IP assays demonstrated that the ACSL4-K500R mutation not only failed to cause a decrease but led to an increase in the overall ubiquitination level of ACSL4. These results collectively indicated that K500 of ACSL4 is not a major site for ubiquitination. Instead, we proposed that SUMOylation at the K500 site induces conformational changes or steric hindrance in ACSL4, thereby inhibiting ubiquitination accessibility to other sites on ACSL4. This hypothesis is supported by prior evidence showing structural antagonism between SUMO and ubiquitin modifications at distinct lysine residues^70,71^.

Inhibition of glycolysis or lactyltransferase may induce more widespread side effects beyond targeting SUMO2-K11la, while the selective permeability of cell membranes also poses a significant obstacle to the intracellular delivery of numerous active molecules. To overcome these challenges of targeting SUMO2-K11la, we developed a SUMO2-K11 peptide conjugated with CPP, a promising class of short peptides with exceptional membrane transduction capabilities^72,73^. Since initial discovery, CPPs have demonstrated utility in the delivery of various cargos (chemotherapeutic drugs, nucleic acids, polypeptides, and proteins) for the treatment of diverse disorders, including cancer, myocardial ischemia, and dermatosis^41,74^. In our study, we found that SUMO2-K11 peptides conjugated with CPPs can enter cells in the absence of any transfection reagent and specifically inhibit SUMO2-K11la to promote ferroptosis in vitro and in vivo.

PDO provides a highly suitable platform for conducting drug sensitivity tests due to its notable ability to recapitulate the histological and molecular characteristics of their corresponding original tissues^75,76^. To further validate our findings, we constructed PDOs from four different LUAD patients and demonstrated that K11-Pep sensitized all these PDOs to ferroptosis as well as CDDP treatment. In this study, we also employed the Cre/LoxP system in C57BL/6 mice harboring the Kras G12D mutation to establish spontaneous lung cancer models, which provide a superior approximation of the endogenous emergence of human tumors, encompassing crucial traits such as tumor heterogeneity and immune microenvironment. The inherent genetic diversity within these models facilitates a more precise representation of tumor progression^54^. The results obtained from the spontaneous lung cancer models corroborated that K11-Pep treatment augmented the efficacy of chemoimmunotherapy, ultimately leading to an improved prognostic outcome. Taken together, these findings suggest that K11-Pep represents a promising therapeutic approach for LUAD treatment.

## Conclusion

Our study elucidates that SUMO2-K11la counteracts ferroptosis by reducing ACSL4 sumoylation in LUAD, and we developed a CPP targeting SUMO2-K11la to promote ferroptosis, which holds significant potential for future therapeutic interventions and clinical applications in LUAD treatment.

## Methods and materials

### Cell lines and reagents

Human cell lines, including A549, PC9, and HEK-293T, were obtained from the Chinese Academy of Sciences Cell Bank and authenticated via Short Tandem Repeat (STR) analysis. The cells were incubated in a humidified environment at 37 °C with 5% CO_2_ in DMEM high glucose medium (KeyGEN BioTECH, Nanjing, China), supplemented with 10% fetal bovine serum (EveryGreen, TIANHANG Biotechnology Co., Ltd, Huzhou, China), 100 μ/mL penicillin, and 0.1 mg/mL streptomycin (Beyotime, Shanghai, China).

FINs, including RSL3 and IKE, LA, NALA, ferroptosis inhibitors, including DFO and Ferr-1, apoptosis inhibitor Z-VAD-FMK, necroptosis inhibitor necrosulfonamide (Necro), CHX, 2-Deoxy-D-glucose (2-DG, dissolved in PBS), sodium oxamate (dissolved in PBS), and Pembrolizumab (anti-PD1, dissolved in normal saline) were bought from MedChemExpress (Princeton, USA). Cisplatin (CDDP, dissolved in PBS), SENP inhibitor N-Ethylmaleimide (NEM), MG-132, Chloroquine, and HDAC1 activator Exifone were sourced from TargetMol (Boston, USA). HDAC1 inhibitor Quisinostat (JNJ-26481585) 2HCl was obtained from Selleck Chemicals (Houston, USA). Hydrochloric acid (HCl, 37% in H_2_O) was bought from Aladdin (Shanghai, China). All compounds, unless otherwise specified, were dissolved in DMSO.

### Metabolite library screening

A human endogenous metabolite library comprising 889 compounds (HY-L030) was obtained from MedChemExpress. LUAD cells were planted into 96-well plates at a density of 2500 cells per well. After 24 h, the cells were pre-incubated with DMSO or the specified metabolites for 12 h, followed by exposure to 2 μM RSL3 for 48 h. Cell viability was assessed using the CellTiter-Lumi™ Luminescent Cell Viability Assay Kit (Beyotime, Shanghai, China) on a microplate reader (SpectraMax iD3, Molecular Devices, San Jose, USA).

### Untargeted metabolomic profiling

Cells were treated with 2 μM RSL3 (24 h) in 9-cm dishes. After PBS washing and liquid nitrogen quenching, cells were harvested in ice-cold methanol: water (4:1) and stored at –80 °C. Samples were homogenized with steel beads in 400 μL methanol-water (4:1, v/v) containing 4 μg/mL L-2-chlorophenylalanine after cryogenic cooling (–40 °C, 2 min). Following grinding, extracts were sonicated (ice-water bath, 10 min), incubated (–40 °C, 30 min), and centrifuged (4 °C, 1200 rpm, 10 min). The supernatant (300 μL) was dried, reconstituted in 300 μL methanol-water (1:4, v/v), vortexed (30 s), sonicated (3 min), and incubated (–40 °C, 2 h). After re-centrifugation, 150 μL supernatant was filtered into LC vials and stored at –80 °C. Quality-control samples comprised pooled aliquots of all extracts.

LC-MS analysis was performed using a UPLC-QE Plus system (Thermo Fisher Scientific, Waltham, USA) with HESI source and ACQUITY UPLC HSS T3 column (1.8 μm, 2.1 × 100 mm). Raw data were processed using Progenesis QI software for peak picking, retention time alignment, and normalization. Metabolite identification utilized HMDB, LipidMaps, Metlin, and in-house databases by matching exact m/z values and MS/MS fragments. Metabolomic analysis was performed by OE Biotech Co., Ltd (Shanghai, China).

### Glycolysis stress test

To quantify the extracellular acidification rate (ECAR) under ferroptosis stress, LUAD cells were seeded in a Seahorse XF96 plate at a density of 30,000 cells per well and allowed to adhere overnight. Following a 24-h treatment with FINs or CDDP, the growth medium was replaced with XF assay medium, and the cells were incubated in a CO_2_-free incubator for 1 h before measurement on a Seahorse XFe24 flux analyzer (Agilent Seahorse Bioscience, Santa Clara, USA). The experimental run involved three sequential injections, and the final concentrations of reagents were as follows: 10 mM glucose, 2 μM oligomycin, and 50 mM 2-DG.

### RNA sequencing

Total RNA extraction was performed using the TRIzol reagent (TIANGEN Biotech, Beijing, China). Sequencing was carried out by OE Biotech Co., Ltd. Subsequently, the raw sequencing data were normalized into Fragments Per Kilobase of exon model per Million mapped fragments (FPKM) format for subsequent analysis.

### LA, GSH, MDA, and 4-HNE detection

The LA abundance, GSH/ GSSG ratio, and MDA content in LAUD cells were measured using the LA Content Assay Kit (Solarbio, Beijing, China), GSH and GSSG Assay Kit (Beyotime), MDA Assay Kit (Beyotime), and Lipid Peroxidation (4-HNE) Assay Kit (Abcam, Cambridge, UK) following the manufacturers’ instructions, respectively. For MDA or GSH/GSSG assays, cells were pre-incubated with or without NALA (5 mM) for 24 h, followed by treatment of RSL3 (2 μM), IKE (10 μM), or CDDP (10 μM) for 8 h.

### Cytotoxicity assays

Cells were seeded in 96-well plates at an appropriate density and allowed to grow overnight. After indicating treatments, cell viability was measured using the Cell Counting Kit-8 (TargetMol) following the manufacturer’s protocols. The absorbance of each well was determined using a microplate reader (SpectraMax iD3, Molecular Devices, San Jose, USA) at 450 nm.

### Lipid peroxidation measurement

Cells were seeded in 12-well plates and allowed to adhere overnight. Subsequently, LUAD cells were pre-incubated with or without NALA (5 mM) for 24 h, followed by treatment with RSL3 (2 μM) for 8 h. After being harvested through trypsinization, the cells were resuspended in fresh media containing 4 μM BODIPY 581/591 C11 fluorescent probe (Thermo Fisher Scientific) at 37 °C for 30 min to allow for probe binding. Unbound dye was subsequently removed through PBS washing, and the levels of lipid peroxidation were measured using an Accuri C6 flow cytometer (BD Biosciences, San Diego, USA). The acquired data were analyzed utilizing FlowJo software (TreeStar, Woodburn, USA).

### Co-IP

The experiment was performed as previously described^77^. The Protein A/G magnetic beads (ThermoFisher Scientific) were incubated with the indicated antibodies overnight at 4 °C and then crosslinked with DSS. The beads were washed with NP-40 Lysis Buffer three times. Cell lysates were incubated with the crosslinked beads for a further overnight at 4 °C. Immunoprecipitated complexes were collected, washed, and then subjected to proteomics profiling or boiled with loading buffer in a dry bath heater for western blot detection.

To investigate the SUMOylation level of endogenous ACSL4, cells were lysed under conditions containing 50 mM NEM to preserve SUMO-conjugated modifications. Subsequently, endogenous ACSL4 was immunoprecipitated utilizing a specific ACSL4 antibody (1:100, ab155282, Abcam) and then subjected to immunoblots. To enhance the detection of SUMOylation or ubiquitination signals, the proteasome inhibitor MG-132 (10 μM) was applied to block substrate protein degradation.

For mapping the AARS1-SUMO2 interaction site, HA-AARS1 (K823A) and His-SUMO2 (D63A) constructs were transfected into HEK293T cells. Protein complexes were precipitated using anti-HA antibody (1:50, #3724, Cell Signaling Technology, Danvers, USA) and subjected to downstream analysis.

### RNA analysis

Total RNA extraction was conducted utilizing the TRIzol reagent, following the manufacturer’s guidelines. Subsequently, the concentration and purity of the extracted RNA were determined with a Denovix DS-11 spectrophotometer (Denovix, Wilmington, USA).

For reverse transcription-quantitative polymerase chain reaction (RT-qPCR), the RNA samples were converted into cDNA using the Hifair II First-Strand cDNA Synthesis Kit (YEASEN, Shanghai, China). This cDNA was then subjected to RT-qPCR utilizing the Hieff qPCR SYBR Green Master Mix (YEASEN) on an ABI QuantStudio 5 real-time PCR system (Thermo Fisher Scientific). Gene expression levels were quantitatively determined using the 2^–ΔΔCT^ method, with normalization to the β-actin gene expression serving as an endogenous control. The list of primers employed in this study was provided in Supplementary Table S3.

### Immunoblot

Cells were lysed in RIPA buffer (MeilunBio, Dalian, China) containing a protease inhibitor and two phosphatase inhibitor cocktails (TargetMol), and maintained on ice for 10 min. After sonication, protein concentrations were determined using a BCA assay kit (YEASEN). Proteins were separated by SDS-PAGE gel (ShareBio, Shanghai, China) and then transferred to PVDF membranes (Millipore, Burlington, USA). Following a one-hour blocking step with 5% non-fat milk at room temperature, membranes were incubated with primary antibodies overnight at 4 °C and then exposed to HRP-conjugated secondary antibodies (1:2500, Beyotime) for 1 h at room temperature. Protein bands were detected using Enhanced Chemiluminescent Reagent (New Cell & Molecular Biotech, Suzhou, China). Details of the primary antibodies were provided in Supplementary Table S3.

### Dot blot assay

SUMO2-K11la peptide (PKEGV-(lactyl)K-TENNDHI) and unmodified SUMO2 peptide (PKEGVKTENNDHINL) were deposited onto PVDF membranes and allowed to dry for 30 min. Subsequently, the blots underwent blocking with 5% non-fat milk for 1 h at room temperature. They were then incubated with the customized SUMO2-K11la antibody for 12 h at 4 °C, followed by incubation with a secondary anti-rabbit IgG antibody for 1 h at room temperature. The blots were detected using Enhanced Chemiluminescent Reagent.

### CRISPR Cas9-mediated gene knock-in

The experimental procedure was conducted as previously reported^26^. In brief, sgRNA targeting the sequence surrounding the mutated nucleotides of SUMO2 was designed utilizing the Crispick tool (https://portals.broadinstitute.org/gppx/crispick/public). Lentiviruses containing sgRNA and Cas9 (puromycin-resistant), a repair template, and an expression plasmid were synthesized by Genechem (Shanghai, China).

LUAD cells were seeded in six-well plates at a density of 4 × 10^5^ cells per well and transfected with siRNAs targeting XRCC4, Lig4, and Ku70 (RIBOBIO, Guangzhou, China) on the subsequent day to inhibit non-homologous end joining, thereby enhancing the efficiency of homology-directed repair. Following a 24-h incubation, LUAD cells were transfected with lentiviruses and screened with puromycin. After 48 h of selection, the cells were diluted to generate clonal cell populations. After approximately two weeks of expansion, genomic DNA was extracted from the selected cell populations, amplified using Taq PCR Master Mix (Sangon Biotech, Shanghai, China), and subjected to Sanger sequencing to assess the knock-in efficiency. Clones harboring the variant of interest were retained for further selection. The sequences of the sgRNA, repair template, and siRNAs used in this study were listed in Supplementary Table S3.

### Immunofluorescence

Cells in the slides were fixed with 4% paraformaldehyde for 15 min. Subsequently, the slides were permeabilized using 0.5% Triton X-100 at room temperature for 20 min. Following permeabilization, goat serum was added to the slides and incubated at room temperature for 30 min to block non-specific binding sites. Next, primary antibodies were applied to the slides and incubated overnight at 4 °C. After overnight incubation, the slides were washed with PBST and then incubated with fluorescent secondary antibodies: anti-mouse or anti-rabbit IgG (Beyotime, 1:300) for 1 h at room temperature. Finally, the slides were sealed with a blocking solution containing an anti-fluorescence quencher and imaged using a fluorescence microscope (Olympus IX71, Tokyo, Japan) or a confocal microscope (Nikon ECLIPSE TI, Tokyo, Japan).

### CHX chase assay

To investigate the half-life of the SUMO2 and ACSL4 proteins, cells were treated with CHX (50 μg/mL) and harvested at defined time points post-treatment. Total cell lysates were collected and then subjected to immunoblot to test the protein levels.

### Transmission electron microscopy

We adopted a standardized protocol for sample preparation, as previously documented^78^. Specifically, cells were cultivated in 6 cm dishes. The NALA-treated group was preconditioned with or without NALA (5 mM) for 24 h and then exposed to DMSO, RSL3 (2 μM), or IKE (10 μM) for 8 h. Following this, cells were fixed with a 2.5% glutaraldehyde solution and thoroughly rinsed three times using PBS. Subsequently, a secondary fixation step was performed using PBS containing 1% osmium acid, with the samples undergoing an additional three washes. The fixed cells then underwent dehydration and embedding procedures, with the final samples being cured in an oven at 60 °C for 48 h. Ultrathin sections were meticulously prepared and subsequently stained with lead citrate and uranyl acetate. Following overnight drying, the sections were examined and imaged using a transmission electron microscope (Hitachi, Tokyo, Japan).

### Patient selection

The resected tumor tissue and clinical data from 140 LUAD patients who underwent surgical resection at the Department of Thoracic Surgery, Zhongshan Hospital, Fudan University, from January 2017 to November 2017 were collected. The surgical procedures were performed by highly skilled thoracic surgeons, and the resected tumors and lymph nodes were evaluated and confirmed by at least two qualified pathologists. Tumor staging was determined according to the eighth edition of the American Joint Committee on Cancer (AJCC) Cancer Staging Manual, using the tumor/lymph node/metastasis (TNM) classification system. This research was conducted following the ethical principles outlined in the Declaration of Helsinki and approved by the Research Ethics Committee of Zhongshan Hospital, Fudan University (approval number: B2022-180R).

### IHC

The formalin-fixed and paraffin-embedded tissue samples were collected. Tissue slides underwent overnight incubation at 65 °C, followed by triple dewaxing in xylene (15 min each) and rehydration in an alcohol gradient. Antigen retrieval was performed using a citrate buffer solution (Sangon Biotech), and endogenous peroxidase activity was blocked at 37 °C for 30 min. The slides were subsequently blocked for 10 min, then incubated with primary antibodies at 4 °C overnight, followed by secondary antibodies at room temperature for 1 h. Staining was achieved using the GTVisionTM III Detection System/Mo&Rb (GeneTech, Shanghai, China) following the manufacturer’s protocol.

To confirm the specificity of SUMO2-K11la antibody, a peptide competition assay was performed. Briefly, the primary antibody was subjected to pre-incubation with a 50-fold molar excess of K11-lactylated SUMO2 peptide for 1 h at room temperature, after which it was applied to IHC sections. Concurrently, parallel control sections were treated solely with the primary antibody, without the peptide competitor. The criterion for specificity validation was a reduction in immunostaining intensity of ≥ 80% in the peptide-blocked sections as compared to the control sections, with quantification performed using ImageJ software.

To quantify IHC results, four random fields of view per slide were assessed for staining intensity and coverage. The staining intensity was graded as 0 (no staining), 1 (weak staining), 2 (moderate staining), or 3 (strong staining), while the staining area was scored from 0 (≤ 5%) to 4 (> 75%) of positive cells. The histological score was calculated by multiplying the staining intensity and coverage area scores, then categorized: negative (−, 0), weak (+, 1–4), moderate (++, 5–8), or strong (+++, 9–12). Scores were dichotomized into low expression (−/+) and high expression (++/+++) groups. The primary antibodies employed in the IHC analysis were listed in Supplementary Table S3.

### 4D label-free quantitative SUMOylated proteomics analysis

Cells were collected, and the 4D label-free quantitative SUMOylated proteomics analysis was performed by Medical Discovery Leader BioLabs (Langfang, China). After quality control, an appropriate amount of protein was taken and incubated with a final concentration of 5 mM DTT at 37 °C for 1 h, followed by returning to room temperature. Lodoacetamide was then added to a final concentration of 10 mM, and the mixture was incubated in the dark at room temperature for 45 min. The sample was diluted fourfold with 25 mM ammonium bicarbonate and incubated with trypsin (protein:trypsin = 50:1) overnight at 37 °C. On the second day, formic acid was added to terminate the enzymatic digestion.

Desalting of the samples was performed using a C18 desalting column. The column was activated with 100% acetonitrile, equilibrated with 0.1% formic acid, and the sample was loaded onto the column. The column was then washed with 0.1% formic acid to remove impurities, and the peptides were eluted with 70% acetonitrile. The eluate was collected and lyophilized.

The peptides were dissolved in immunoaffinity purification (IAP) buffer and centrifuged at a low temperature to remove precipitates. Commercial antibody-conjugated beads were washed twice with PBS and twice with IAP buffer, followed by centrifugation. The beads were then subjected to rotational incubation for 2 h. The beads were washed twice with IAP buffer and three times with ice-cold water, with repeated centrifugation steps. Subsequently, 0.15% TFA was added, and the mixture was incubated at room temperature for 10 min with gentle inversions every 2 min. After centrifugation, the supernatant was collected and subjected to LC-MS detection and annotated using the Homo sapiens SP database.

### Lipidomics

Cells were washed three times with PBS and detached by trypsinization. After centrifugation, the pellet was resuspended in PBS for cell counting. Cells were recentrifuged, flash-frozen in liquid nitrogen for 30 s, and stored on dry ice for shipment. Samples were mixed with 100 μL water and 480 μL extraction solvent, vortexed, and sonicated on ice. After centrifugation, 250 μL supernatant was collected. The extraction was repeated twice with fresh solvent (250 μL each), combining all supernatants. The pooled supernatant was lyophilized at 37 °C, reconstituted in 150 μL solvent, vortexed, sonicated, and centrifuged before LC-MS/MS analysis. Chromatographic separation was performed on a Nexera LC-40 UPLC system using an ACQUITY UPLC BEH C18 column (2.1 × 100 mm) with mobile phase A (60% acetonitrile/40% water, 10 mM ammonium acetate, 0.1% acetic acid) and phase B (10% acetonitrile/90% isopropanol, 10 mM ammonium acetate, 0.1% acetic acid) at 0.3 mL/min. Samples were analyzed by SCIEX Triple Quad 7500 mass spectrometer in MRM mode. Optimal precursor-product ion pairs and collision energies were predetermined for each lipid analyte. The lipidomics analysis was conducted by Biotree Biomedical Technology Co., Ltd (Shanghai, China).

### PLA

The PLA was performed using the Duolink In Situ PLA kit (Sigma, Saint Louis, USA) to detect the interaction between SUMO2 and ACSL4. Briefly, cells were seeded on coverslips in 24-well plates and cultured overnight. After PBS washing, cells were fixed with 4% paraformaldehyde, permeabilized with 0.1% Triton X-100, and blocked with 3% BSA. Primary antibodies, including anti-SUMO2 (Rabbit, 1:200, #AY9566, Abways, Shanghai, China) and anti-ACSL4 (Mouse, 1:200, #66617-1-Ig, Proteintech, Wuhan, China), were incubated overnight at 4 °C. For PLA, species-specific PLA probes (anti-mouse PLUS and anti-rabbit MINUS) were hybridized at 37 °C for 1 h, followed by ligation (30 min) and amplification (100 min) steps. Nuclei were stained with DAPI. Each pair of proteins generated a spot that was visualized using a confocal microscope (Nikon ECLIPSE TI).

### siRNA transfection

siRNAs targeting corresponding genes were obtained from RIBOBIO Corporation and transfected into cells using the lipo8000 reagent (Beyotime) and Opti-MEM medium (Gbico, Carlsbad, USA), following the manufacturer’s guidelines. The sequences of corresponding siRNAs were listed in Supplementary Table S3.

### Molecular docking

Protein sequences of SUMO2, AARS1, and HDAC1 were retrieved from the UniProt database (https://www.uniprot.org/) and subjected to structural interaction prediction using AlphaFold3 (https://golgi.sandbox.google.com/). A comprehensive investigation of the residue-specific interactions between these proteins was performed and visualized using ChimeraX software (San Francisco, USA).

### In vitro lactylation assay

The recombinant HA-AARS1 (5 μM) and His-SUMO2/His-SUMO2-K11R (10 μM, synthesized by Sangon Biotech) were incubated in a reaction buffer consisting of 100 mM HEPES, 100 mM MgCl_2_, 10 mM KCl, 5 mM ATP, and 10 mM LA. The incubation was carried out at 37 °C for 1 h, and the products were subjected to immunoblot analysis for further characterization.

### Peptide synthesis

All peptides were synthesized by Sangon Biotech and purified by high-pressure liquid chromatography to a purity greater than 95%. All the amino acids in the peptides were in D isoforms. The peptides were dissolved in PBS for in vitro and in vivo use.

### Establishment of PDOs and drug sensitivity tests

The experiment was conducted as previously reported^79^. Fresh LUAD tumor specimens were dissected into small fragments, washed three times with ice-cold PBS containing 0.3 mg/mL streptomycin and 300 units/mL penicillin, and subsequently digested using an organoid digestive solution (D1Med, Hangzhou, China). Following sequential filtration and centrifugation steps, the resultant cell precipitate was resuspended in Matrigel (Absin, Shanghai, China) at a 1:25 ratio and plated into 24-well plates. After allowing the Matrigel to solidify at 37 °C for 10 min, 500 μL of human LUAD-PDO culture medium (D1Med) was added to each well to support the growth of the PDOs. For histological examination, PDOs were collected and fixed in 4% paraformaldehyde, embedded in agarose and paraffin, sectioned, and subjected to standard H&E staining steps, including deparaffinization, dehydration, and staining. For drug sensitivity tests, PDOs were seeded in 96-well plates and treated with specified compounds. Cell viability was then assessed using the CellTiter-Lumi Luminescent 3D Cell Viability Assay Kit (Beyotime).

### Animal experiment

This research was performed in accordance with the guidelines of the Research Ethics Committee of Zhongshan Hospital, Fudan University. The mice were housed in pathogen-free laminar flow cabinets and treated humanely.

For cell-derived xenograft models, each BALB/c nude mouse (GemPharmatech, Beijing, China) was subcutaneously inoculated in the right flank with 2 × 10^6^ A549 cells suspended in 100 μL PBS. Upon the tumors reaching an approximate volume of 50 mm^3^, the mice were randomly allocated into three groups (*n* = 5 per group): Ctrl, K11-Pep (5 mg/kg), and K11R-Pep (5 mg/kg). Each group was intraperitoneally administered with either IKE (30 mg/kg), CDDP (3 mg/kg), or normal saline every three days, for a total of six administrations. Notably, none of the treatments significantly influenced the body weight of the mice. Tumor sizes were recorded weekly using vernier calipers, and the mice were euthanized after 4 weeks. Tumor volume was computed using the following formula: (length × width^2^)/2.

As for the spontaneous lung cancer model, six-week-old male C57BL/6 mice carrying a Cre-LoxP-mediated G12D mutant Kras (Shanghai Model Organisms Center, Shanghai, China) were intranasally administered with 5 × 10^10^ copies of Scgb1a1-Cre adeno-associated virus (Genechem). This viral vector system effectively mediated the excision of stop elements, resulting in the activation of oncogenic KRAS expression and subsequent lung tumorigenesis. After two months, the mice underwent CT scanning to detect tumor formation. The mice with confirmed lung cancer were intraperitoneally injected with CDDP (3 mg/kg) in combination with anti-PD1 (100 μg per mouse) or the isotype control every three weeks for a total of four administrations. Additionally, the mice received intraperitoneal injections of either K11-Pep (5 mg/kg) or K11R-Pep (5 mg/kg) every three days for 22 times. CT scans were conducted (‌Rigaku Corporation, Tokyo, Japan) to assess tumor progression.

### Single-cell sequencing and data processing

Single-cell RNA sequencing data were obtained from 29 samples, comprising 12 normal lung tissues and 17 LUAD tissues, as previously described^80^. Following data acquisition, rigorous quality control measures were applied, followed by dimensionality reduction and unsupervised clustering analyses to classify cell types and assess gene expression patterns.

### Multiplex IHC

The tissue slides were prepared according to the aforementioned IHC procedures. After blocking steps, the slides were incubated overnight at 4 °C with primary antibodies, followed by PBS washes to remove unbound antibodies. Secondary antibodies were applied for 1 h at room temperature, followed by treatment with Tyramide signal amplification (TSA) agents (Servicebio, Wuhan, China) for 10 min. To strip the bound primary and secondary antibodies, sections were immersed in citrate buffer (pH 6.0) and heated in a microwave for 10 min at boiling conditions. The blocking step was repeated, after which the second round of primary antibodies was applied, followed by incubation with secondary antibodies and TSA reagents. Nuclei were visualized with DAPI (Servicebio). TSA reagents used in this study included iF488-Tyramide (1:500, Green), iF546-Tyramide (1:500, Red), and iF647-Tyramide (1:500, Yellow). A detailed list of primary antibodies used was provided in Supplementary Table S3.

### Flow cytometry analysis

To quantify the infiltration levels of CD8^+^ and CD4^+^ T cells in spontaneous lung cancer models, fresh tumor tissues were dissociated with the Mouse Tumor Dissociation Kit (Miltenyi Biotec, Germany) and filtered through a 70-μm cell strainer. Erythrocytes were lysed with RBC lysis buffer (Beyotime), and dead cells were removed via magnetic bead-based depletion (Miltenyi Biotec). The viable cells were blocked with 2% TruStain FcX™ (anti-mouse CD16/32) Antibody (BioLegend, San Diego, USA) for 10 min on ice, then stained with Zombie Aqua Fixable Viability Kit (BioLegend, #423101) and indicating antibodies, including FITC anti-mouse CD45 (BioLegend, #103107), APC anti-mouse CD4 (BioLegend, #100515), and PE anti-mouse CD8a (BioLegend, #100707) under dark conditions. Following staining, the cells were washed with PBS and analyzed using a FACS Aria III flow cytometer (BD Biosciences) equipped with a 488-nm laser. The data were then processed and analyzed using FlowJo software.

### Quantification and statistical analysis

The results were presented as mean ± standard deviation. Statistical analysis and data visualization were executed using R (Version 4.1.1, R Foundation for Statistical Computing, Vienna, Austria) and GraphPad Prism 9 (GraphPad Software, La Jolla, USA). Either an unpaired Student’s *t*-test or one-way ANOVA with Bonferroni correction was employed to assess continuous variables depending on the context. IC_50_ was calculated with three-parameter dose-response curve models. All statistical tests were two-tailed, and significance was defined as a *p*-value of less than 0.05.

## Supplementary information

Supplementary Data S1

Supplementary Data S2

Supplementary Fig. S1

Supplementary Fig. S2

Supplementary Fig. S3

Supplementary Fig. S4

Supplementary Fig. S5

Supplementary Fig. S6

Supplementary Fig. S7

Supplementary Fig. S8

Supplementary Tab. S1

Supplementary Tab. S2

Supplementary Tab. S3

## Data Availability

The original data generated in this work can be directed to the corresponding author for rational reasons.
