# Supplementary material for: Ferroptosis-induced SUMO2 lactylation counteracts ferroptosis by enhancing ACSL4 degradation in lung adenocarcinoma
Source: Cell Discov. 2025 Oct 7;11:81. doi: 10.1038/s41421-025-00829-6 (PMC12504568; doi:10.1038/s41421-025-00829-6)
Supplement: Supplementary file 3 — Supplementary Fig. S1 [file 41421_2025_829_MOESM3_ESM.pdf]

Supplementary figures

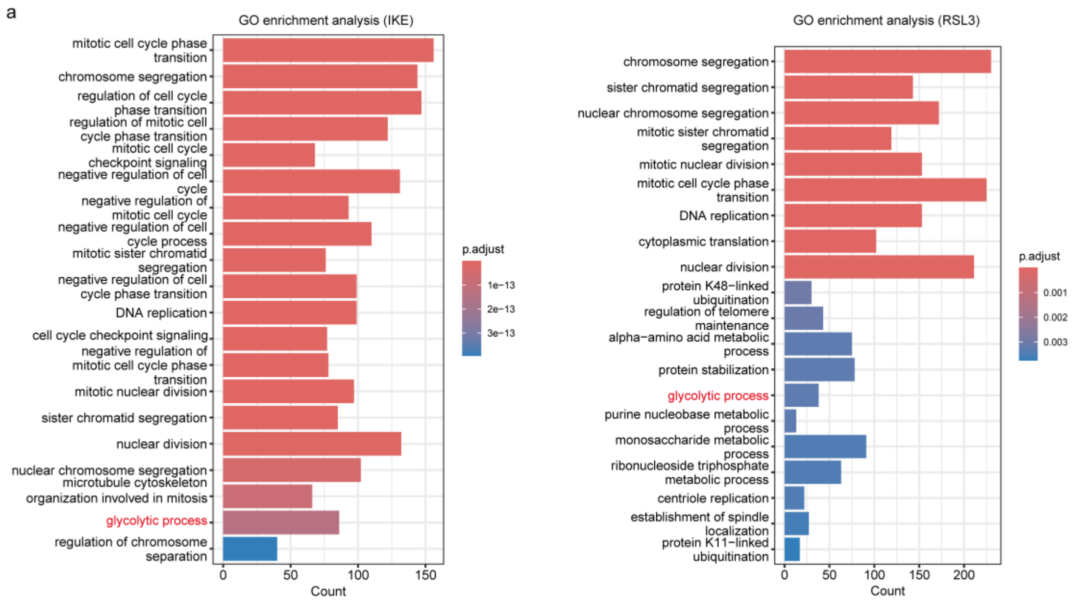

Supplementary Fig. S1 a-b Gene ontology (GO) enrichment analysis showed that the differential genes were enriched in the glycolytic process. The differential genes (|$\log_2$FC| > 0.25, adjusted *p*-value <0.05) were generated using the transcriptomic data of PC9 cells treated with DMSO, IKE (a), or RSL3 (b).
