# Supplementary material for: Ferroptosis-induced SUMO2 lactylation counteracts ferroptosis by enhancing ACSL4 degradation in lung adenocarcinoma
Source: Cell Discov. 2025 Oct 7;11:81. doi: 10.1038/s41421-025-00829-6 (PMC12504568; doi:10.1038/s41421-025-00829-6)
Supplement: Supplementary file 4 — Supplementary Fig. S2 [file 41421_2025_829_MOESM4_ESM.pdf]

a

Lactylation intensity changes of the Top 3 proteins and sites after indicating treatment

Fold change of intensity	LA / Ctrl	RSL3 / Ctrl	CDDP / Ctrl
SUMO2-K11	9.77	1.99	2.56
EDF1-K23	3.54	1.85	1.02
TMSB10-K26	5.26	1.55	2.86

b

Lactylome results of Non-small cell lung cancer tissues [Chen J, et al. *Adv Sci (Weinh)*. 2024.]

Protein accession	Position	Gene name	PEP	Score	Modified sequence	Charge	Mass error [ppm]
P61956	7	**SUMO2**	0.0009	87.942	ADEK(0.006)PK(0.994)EGVK	2	0.9288
P61956	7	**SUMO2**	0.0016	103.870	ADEKPK(1)EGVK	2	-1.395
P55854	7	SUMO3	0.0047	66.664	SEEK(0.046)PK(0.954)EGVK	2	0.177
P61956	11	**SUMO2**	0.0005	54.270	EGVK(1)TENNDHINLK	2	0.0575
P61956	42	**SUMO2**	0.0120	74.269	HTPLSK(1)LMK	2	-0.101

Lactylome results of hepatocellular carcinoma tissues (Yang Z, et al. *Nat Metab*. 2023.)

Protein accession	Position	Gene name	PEP	Score	Modified sequence	Charge	Mass error [ppm]
P61956	42	**SUMO2**	0.006168	83.623	HTPLSK(1)LMK	2	0.28656
P55854	7	SUMO3	0.009205	94.66	SEEK(0.002)PK(0.998)EGVK	2	-8.1547
P61956	7	**SUMO2**	8.22E-05	120.65	ADEKPK(1)EGVK	2	-2.1755
P61956	11	**SUMO2**	1.18E-13	140.57	EGVK(1)TENNDHINLK	3	-0.45647

c

SUMO2-K11 surrounding sequence

Homo sapiens MADEKPKEGVKTENNDHINLKVAGQDGSVVQFKIKRHTP

Mus musculus MADEKPKEGVKTENNDHINLKVAGQDGSVVQFKIKRHTP

Rattus norvegicus MADEKPKEGVKTENNDHINLKVAGQDGSVVQFKIKRHTP

d

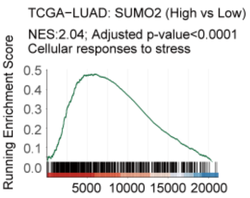

e

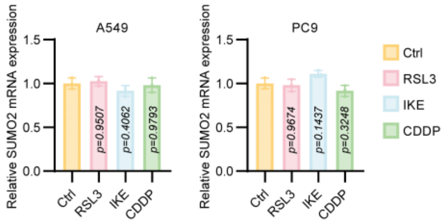

f

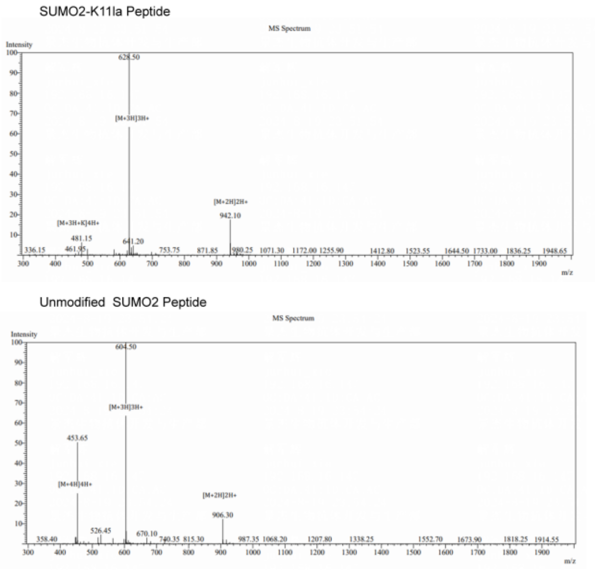

g

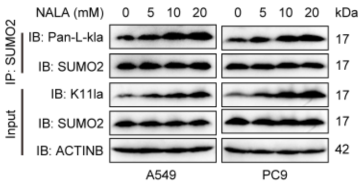

Supplementary Fig. S2 a Lactylation intensity changes of the top three proteins/lysine sites in A549 cells treated with LA (20 mM), RSL3 (2 μM), or CDDP (10 μM). **b** The identified lactylated sites of SUMO2 and its paralogue SUMO3 in previously reported studies. **c** SUMO2-K11 sequence alignment across different species. **d** Gene set enrichment analysis (GSEA) of TCGA-LUAD transcriptomic data linked SUMO2 expression to stress-response pathways. **e** SUMO2 mRNA levels remained unchanged upon stimulation of RSL3 (2 μM), IKE (10 μM), or CDDP (10 μM) treatment for 24 h. **f** Mass Spectrometry identification of lactylation-modified and unmodified SUMO2-

K11 peptides. **g** Co-IP assays coupled with Pan-L-Kla antibody demonstrated a dose-dependent increase in both global lactylation and K11-site-specific lactylation upon NALA treatment. Data were analyzed by one-way ANOVA and were presented by mean ± SD.
