# Supplementary material for: Ferroptosis-induced SUMO2 lactylation counteracts ferroptosis by enhancing ACSL4 degradation in lung adenocarcinoma
Source: Cell Discov. 2025 Oct 7;11:81. doi: 10.1038/s41421-025-00829-6 (PMC12504568; doi:10.1038/s41421-025-00829-6)
Supplement: Supplementary file 5 — Supplementary Fig. S3 [file 41421_2025_829_MOESM5_ESM.pdf]

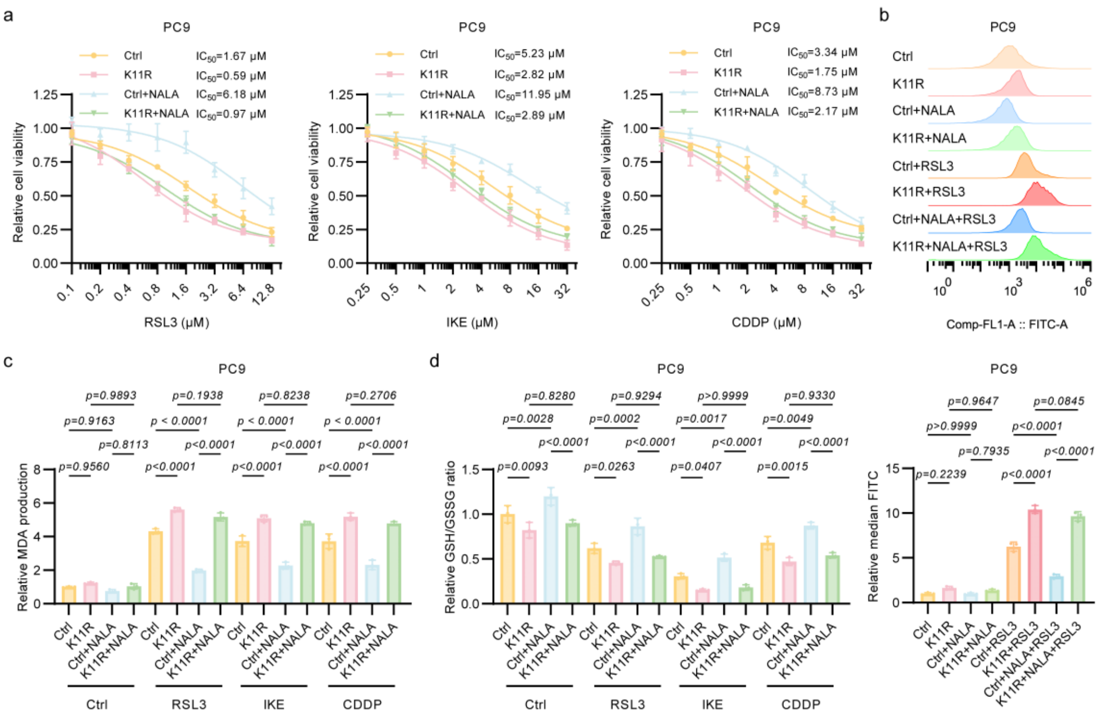

Supplementary Fig. S3 a Relative viability of PC9 Ctrl and K11R cells following treatment with varying concentrations of RSL3 (48 h), IKE (72 h), or CDDP (72 h) with or without concurrent incubation with NALA (5 mM). **b-d** Quantification of ferroptosis-associated biomarkers demonstrated the ferroptosis-promoting effect of K11R mutation: lipid-ROS were measured via BODIPY-C11 fluorescence probes (b), MDA levels were assessed by thiobarbituric acid assay (c), and cellular redox status was determined by GSH/GSSG ratio (d). Data were analyzed by one-way ANOVA and were presented by mean ± SD.
