# Supplementary material for: Ferroptosis-induced SUMO2 lactylation counteracts ferroptosis by enhancing ACSL4 degradation in lung adenocarcinoma
Source: Cell Discov. 2025 Oct 7;11:81. doi: 10.1038/s41421-025-00829-6 (PMC12504568; doi:10.1038/s41421-025-00829-6)
Supplement: Supplementary file 6 — Supplementary Fig. S4 [file 41421_2025_829_MOESM6_ESM.pdf]

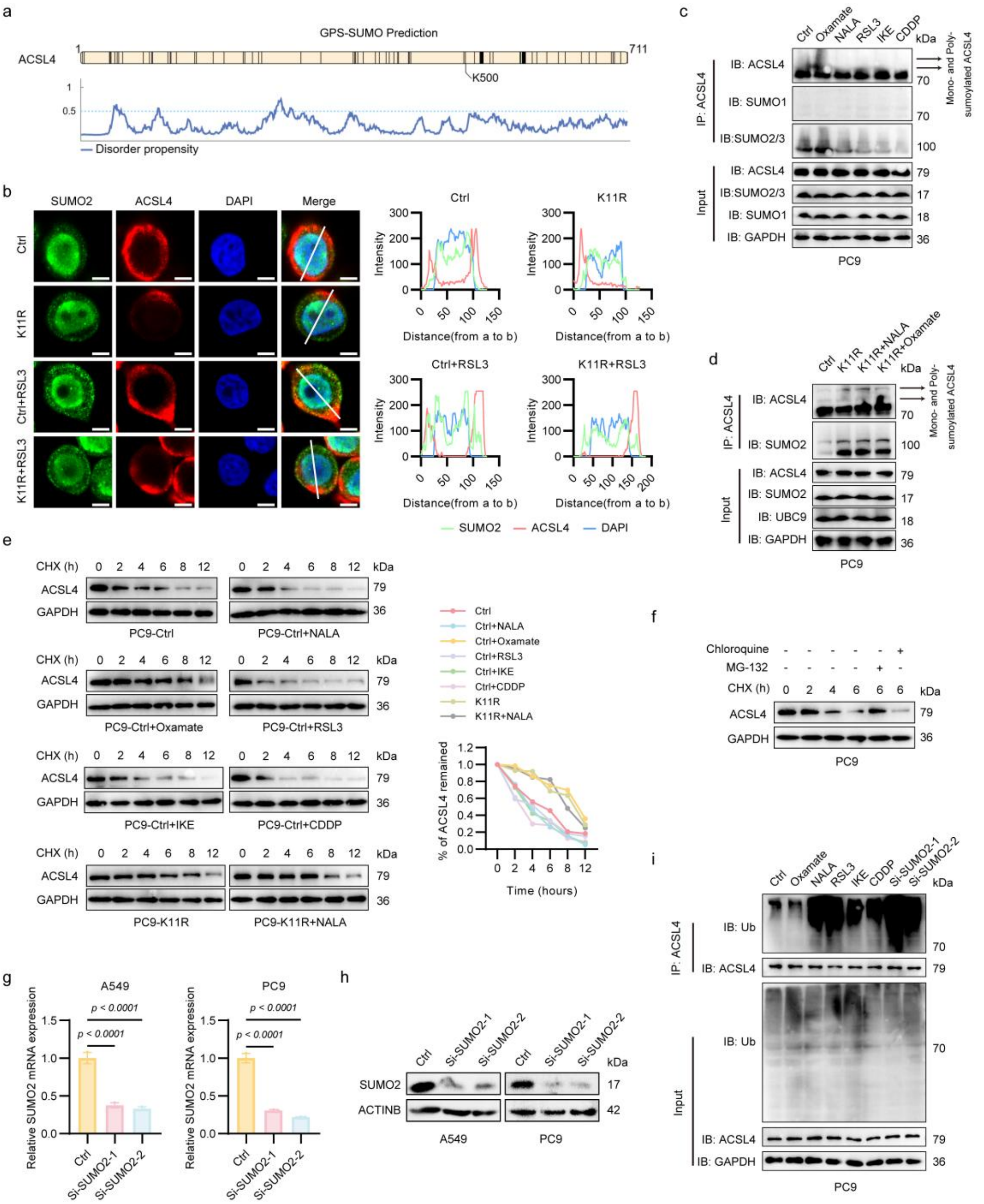

Supplementary Fig. S4 a In silico prediction of the ACSL4-K500 sumoylation motif using the GPS-SUMO algorithm. **b** Immunofluorescence confirmed spatial co-localization of SUMO2 (green) and ACSL4 (red) in LUAD cells, but remained unaltered under SUMO2-K11R mutation or FIN treatment. Nuclei counterstained with DAPI (blue). Scale bars, 5 µM. **c** Co-IP assays demonstrated suppression of SUMO2/3-mediated ACSL4 sumoylation by NALA or FINs, and its augmentation by sodium oxamate. **d** SUMO2-K11R mutation abolished lactylation-dependent regulation and hyper-sumoylated ACSL4 in PC9 cells. **e** CHX chase assays showed accelerated ACSL4 degradation upon NALA/FIN treatment, reversed by sodium oxamate or SUMO2-K11R mutation in PC9 cells. **f** Proteasomal inhibitor MG132 (10 µM), but not lysosomal inhibitor chloroquine (20 µM), rescued ACSL4 degradation in PC9 cells. **g-h** PCR (G) and Western blot (H) showed the construction of SUMO2-knockdown LUAD cells. **i** Ubiquitination assays indicated increased polyubiquitination of ACSL4 following NALA/FIN treatment or

SUMO2 knockdown, contrasting with sodium oxamate-mediated suppression in PC9 cells. Data were analyzed by one-way ANOVA and were presented by mean ± SD.
