# Supplementary material for: Ferroptosis-induced SUMO2 lactylation counteracts ferroptosis by enhancing ACSL4 degradation in lung adenocarcinoma
Source: Cell Discov. 2025 Oct 7;11:81. doi: 10.1038/s41421-025-00829-6 (PMC12504568; doi:10.1038/s41421-025-00829-6)
Supplement: Supplementary file 7 — Supplementary Fig. S5 [file 41421_2025_829_MOESM7_ESM.pdf]

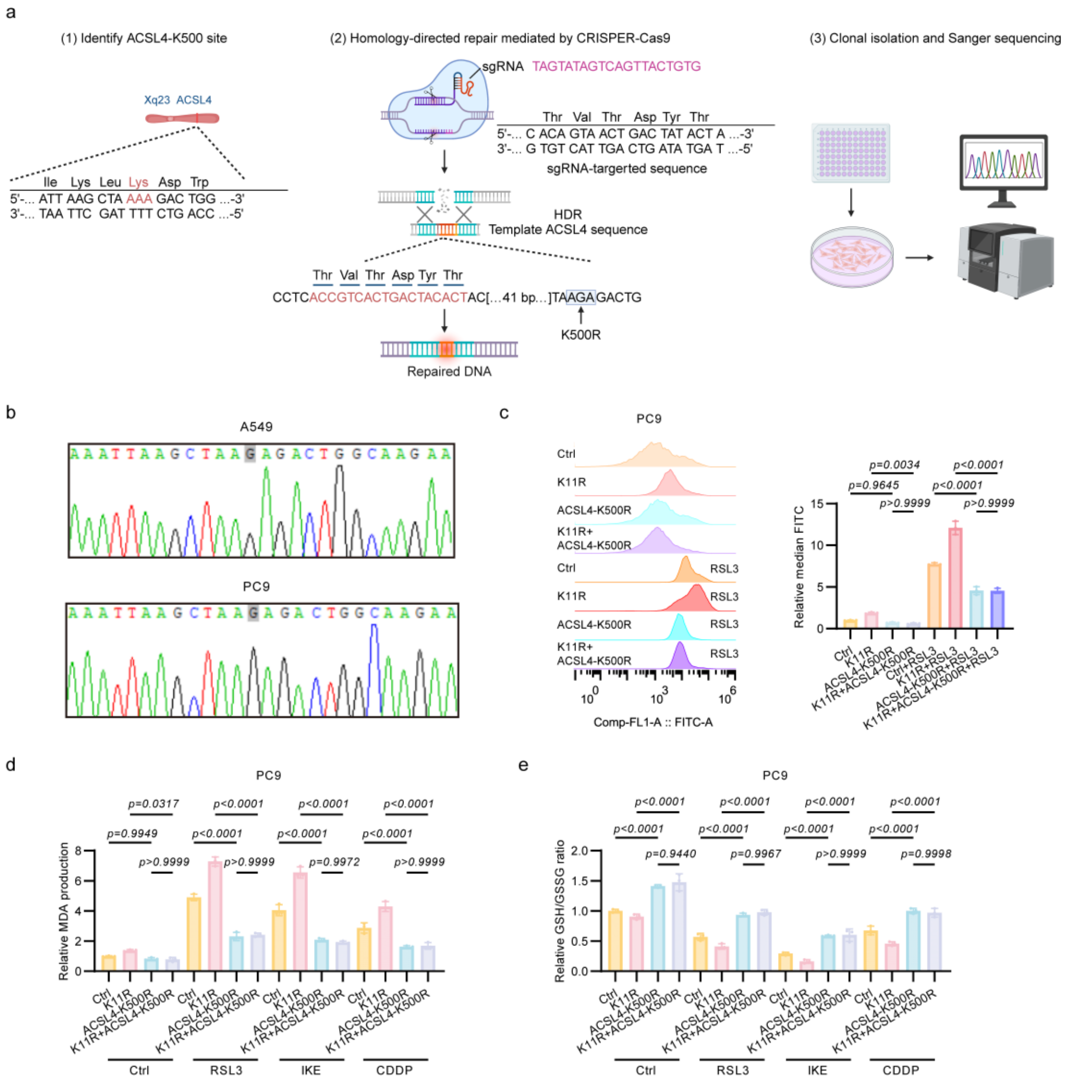

Supplementary Fig. S5 a Flow diagram illustrated the construction of ACSL4-K500R lactylation-defective LUAD cells via the CRISPR-Cas9 system-mediated homology-directed repair. **b** Sanger sequencing demonstrated the introduction of ACSL4-K500R mutation in A549 and PC9 cells, separately. **c-e** K500R mutants conferred ferroptosis resistance and abolished the ferroptosis-promoting effect of K11R, as evidenced by lipid peroxidation detection (c), MDA (d) and GSH/GSSG measurements (e). Data were analyzed by one-way ANOVA and were presented by mean ± SD.
