# Supplementary material for: Ferroptosis-induced SUMO2 lactylation counteracts ferroptosis by enhancing ACSL4 degradation in lung adenocarcinoma
Source: Cell Discov. 2025 Oct 7;11:81. doi: 10.1038/s41421-025-00829-6 (PMC12504568; doi:10.1038/s41421-025-00829-6)
Supplement: Supplementary file 8 — Supplementary Fig. S6 [file 41421_2025_829_MOESM8_ESM.pdf]

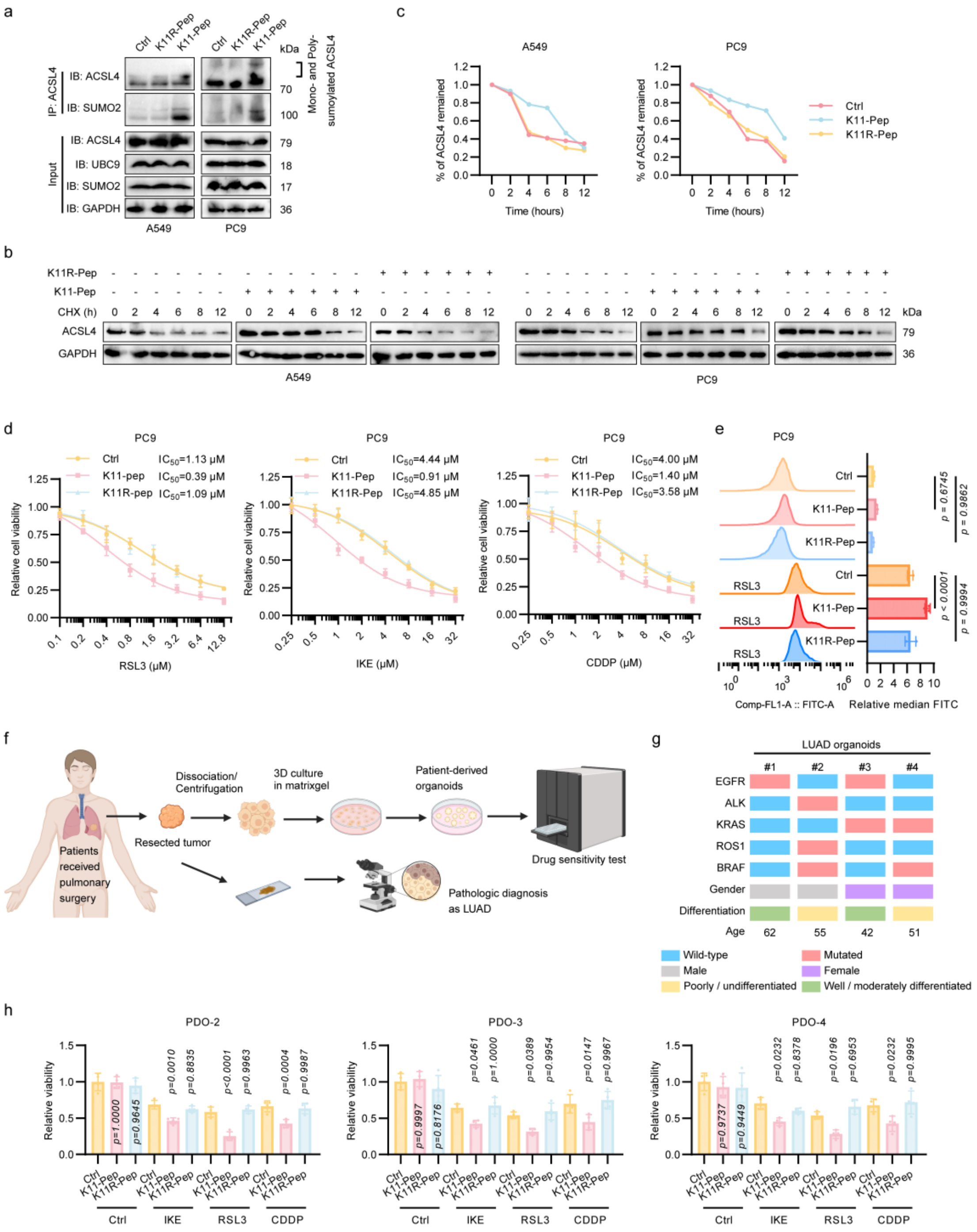

Supplementary Fig. S6 a-c Western blot analysis demonstrated that K11-Pep (but not K11R-Pep) increased ACSL4 sumoylation (a) and attenuated its degradation (b-c). **d-e** K11-Pep potentiated ferroptotic cell death, evidenced by elevated cytotoxicity (d) and lipid peroxidation (e) in PC9 cells. **f** Schematic representation of the establishment procedure for LUAD-PDOs. **g** Clinical information of PDOs from LUAD patients. **h** 3D viability assays revealed K11-Pep-mediated sensitization of PDOs to FINs and CDDP. PDOs were treated with IKE (20 μM), RSL3 (10 μM), or CDDP (30 μM) for 120 h, with or without co-incubation of K11-Pep/ K11R-Pep (10 μM). Data were analyzed by one-way ANOVA and were presented by mean ± SD.
