# Supplementary material for: Ferroptosis-induced SUMO2 lactylation counteracts ferroptosis by enhancing ACSL4 degradation in lung adenocarcinoma
Source: Cell Discov. 2025 Oct 7;11:81. doi: 10.1038/s41421-025-00829-6 (PMC12504568; doi:10.1038/s41421-025-00829-6)
Supplement: Supplementary file 9 — Supplementary Fig. S7 [file 41421_2025_829_MOESM9_ESM.pdf]

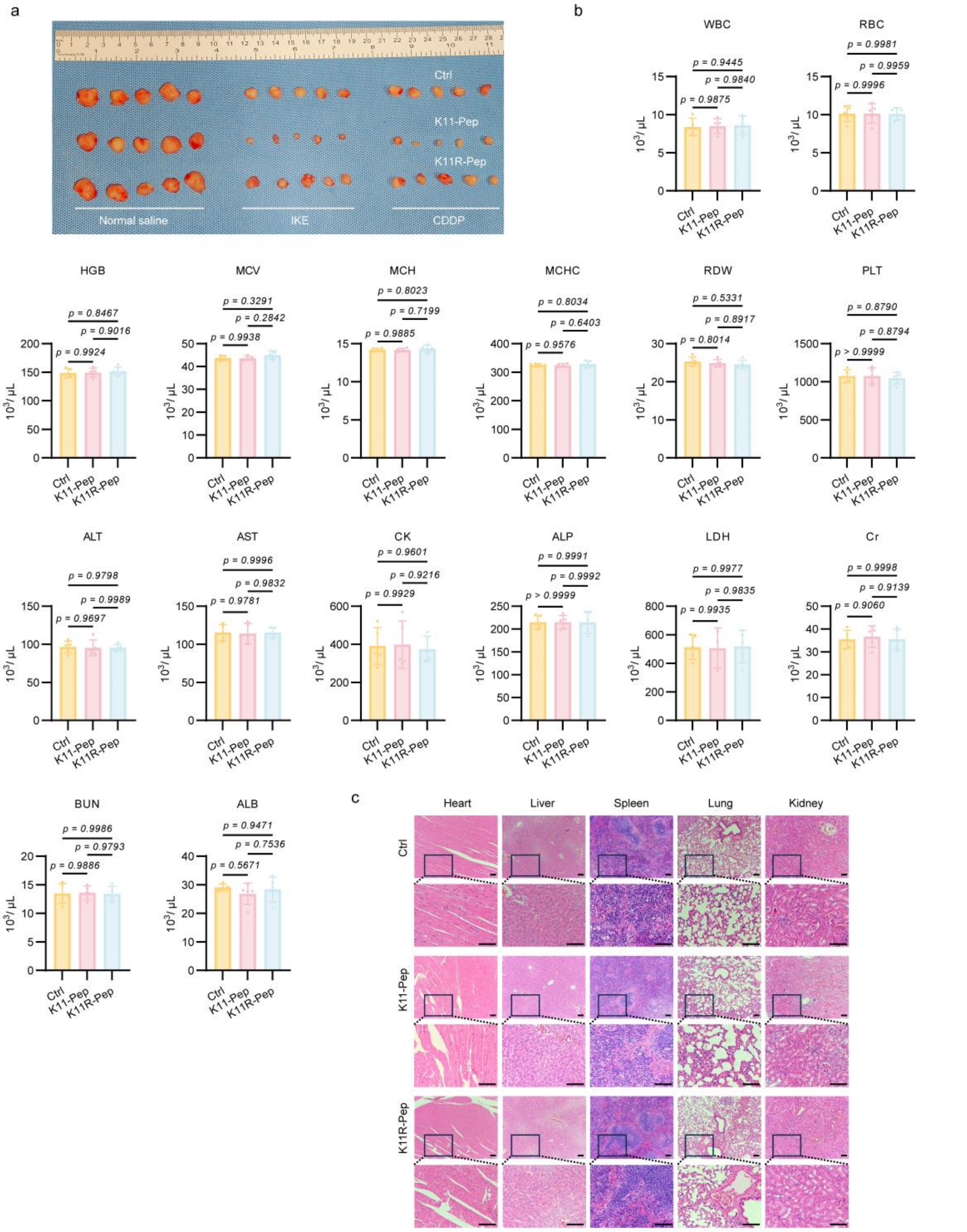

Supplementary Fig. S7 a The resected tumors of different groups. **b** Comparative hematological profiling of control, K11-Pep, and K11R-treated mice showed no significant perturbations in blood parameters. **c** Representative HE-stained sections of major organs (heart, liver, spleen, lung, and kidney) from Pep-treated and control mice demonstrated preserved tissue integrity. Scale bars, 100 μm. Data were analyzed by one-way ANOVA and were presented by mean ± SD.
