# Supplementary material for: Ferroptosis-induced SUMO2 lactylation counteracts ferroptosis by enhancing ACSL4 degradation in lung adenocarcinoma
Source: Cell Discov. 2025 Oct 7;11:81. doi: 10.1038/s41421-025-00829-6 (PMC12504568; doi:10.1038/s41421-025-00829-6)
Supplement: Supplementary file 10 — Supplementary Fig. S8 [file 41421_2025_829_MOESM10_ESM.pdf]

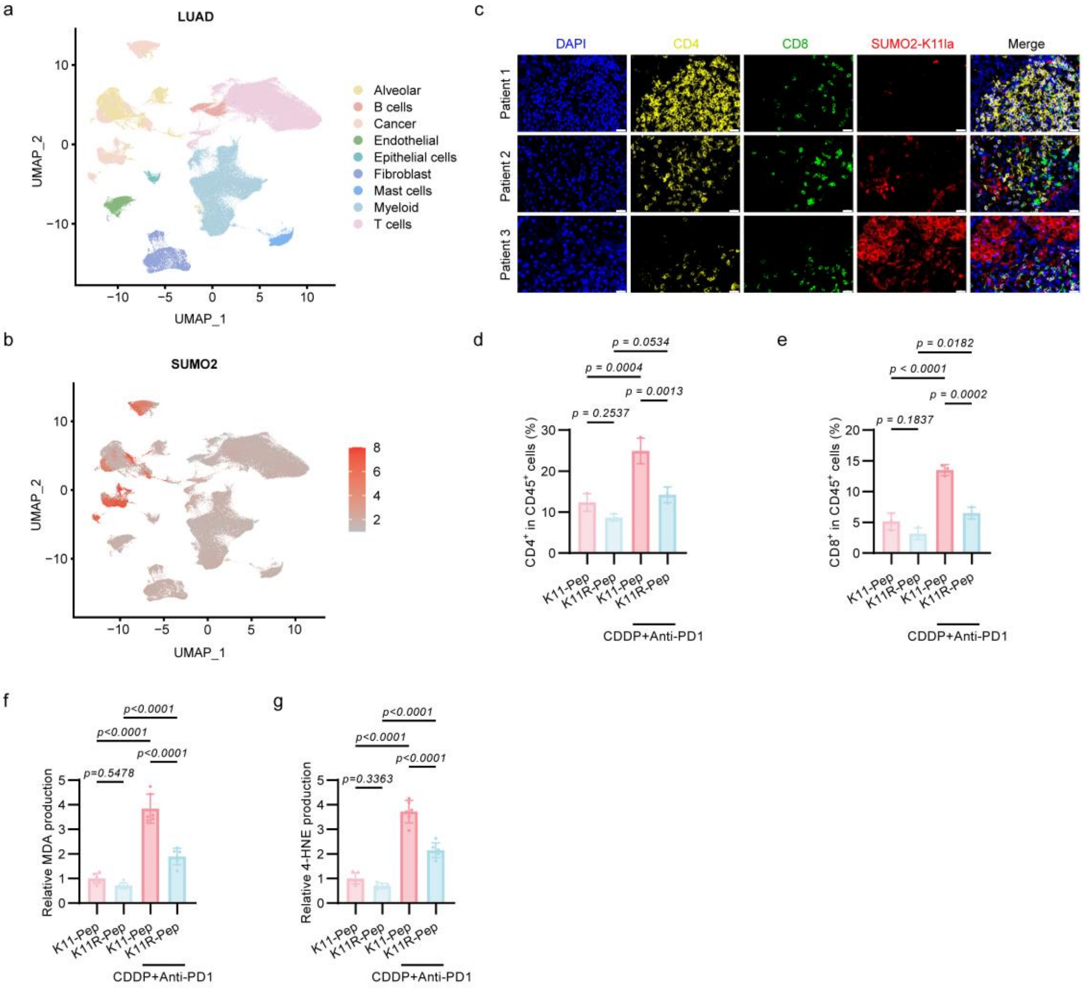

Supplementary Fig. S8 a UMAP visualization delineated transcriptional heterogeneity across 12 normal lung and 17 LUAD clinical specimens, annotated by major cell types. **b** Single-cell RNA sequencing data showed that SUMO2 is predominantly expressed in tumor cells. **c** Multiplex immunohistochemical (mIHC) staining of LUAD patient samples demonstrated spatial segregation of SUMO2-K11la from $CD4^+$ and $CD8^+$ T cell infiltrates. Scale bars, 100 μm. **d-e** Flow cytometry analysis of $CD8^+$ and $CD4^+$ T cells across experimental cohorts. **f-g** Measurement of MDA (f) and 4-HNE (g) in resected tumors from the spontaneous lung cancer model. Data were analyzed by one-way ANOVA and were presented by mean ± SD.
