# Supplementary material for: Ferroptosis-induced SUMO2 lactylation counteracts ferroptosis by enhancing ACSL4 degradation in lung adenocarcinoma
Source: Cell Discov. 2025 Oct 7;11:81. doi: 10.1038/s41421-025-00829-6 (PMC12504568; doi:10.1038/s41421-025-00829-6)
Supplement: Supplementary file 11 — Supplementary Tab. S1 [file 41421_2025_829_MOESM11_ESM.pdf]

Supplementary Table S1

Clinical information of 140 LUAD patients

**SUMO2-K11la expression**	**High (*N=75*)**	**Low (*N=65)***	**P-value**
**Age**			0.990
<60	30 (40.0%)	27 (41.5%)	
>60	45 (60.0%)	38 (58.5%)	
**Stage**			0.303
I+II	52 (69.3%)	51 (78.5%)	
III+IV	23 (30.7%)	14 (21.5%)	
**Gender**			0.470
Female	46 (61.3%)	35 (53.8%)	
Male	29 (38.7%)	30 (46.2%)	
**TNM-T**			0.108
1	43 (57.3%)	33 (50.8%)	
2	13 (17.3%)	22 (33.8%)	
3	15 (20.0%)	7 (10.8%)	
4	4 (5.33%)	3 (4.62%)	
**TNM-N**			0.066
0	35 (46.7%)	43 (66.2%)	
1	27 (36.0%)	14 (21.5%)	
2	13 (17.3%)	8 (12.3%)	
**TNM-M**			/
0	75 (100%)	65 (100%)	
