# Supplementary material for: Ferroptosis-induced SUMO2 lactylation counteracts ferroptosis by enhancing ACSL4 degradation in lung adenocarcinoma
Source: Cell Discov. 2025 Oct 7;11:81. doi: 10.1038/s41421-025-00829-6 (PMC12504568; doi:10.1038/s41421-025-00829-6)
Supplement: Supplementary file 12 — Supplementary Tab. S2 [file 41421_2025_829_MOESM12_ESM.pdf]

Supplementary Table S2

Clinical information of 45 LUAD patients who received cisplatin-based chemotherapy

SUMO2-K11la expression	High (*N=28*)	Low (*N=17*)	P-value
**Age**			0.142
<60	6 (21.4%)	8 (47.1%)	
>60	22 (78.6%)	9 (52.9%)	
**Stage**			0.770
I+II	9 (32.1%)	7 (41.2%)	
III+IV	19 (67.9%)	10 (58.8%)	
**Gender**			0.068
Female	19 (67.9%)	6 (35.3%)	
Male	9 (32.1%)	11 (64.7%)	
**TNM-T**			0.606
1	10 (35.7%)	4 (23.5%)	
2	6 (21.4%)	6 (35.3%)	
3	9 (32.1%)	4 (23.5%)	
4	3 (10.7%)	3 (17.6%)	
**TNM-N**			0.655
0	4 (14.3%)	4 (23.5%)	
1	15 (53.6%)	9 (52.9%)	
2	9 (32.1%)	4 (23.5%)	
**TNM-M**			/
0	28 (100%)	17 (100%)	
