# Supplementary material for: Ferroptosis-induced SUMO2 lactylation counteracts ferroptosis by enhancing ACSL4 degradation in lung adenocarcinoma
Source: Cell Discov. 2025 Oct 7;11:81. doi: 10.1038/s41421-025-00829-6 (PMC12504568; doi:10.1038/s41421-025-00829-6)
Supplement: Supplementary file 13 — Supplementary Tab. S3 [file 41421_2025_829_MOESM13_ESM.pdf]

Supplementary Table S3

Primary antibodies for Western blot	Producer	Source	ID/ Number	Dilution
ACTINB (HRP Conjugated)	Servicebio	/	ZB15001-HRP-100	1:3000
HDAC1	Proteintech	Rabbit	10197-1-AP	1:5000
SUMO2	Abways	Rabbit	AY9566	1:2000
SUMO2/3	Proteintech	Rabbit	11251-1-AP	1:2000
SUMO1	Abways	Rabbit	CY5242	1:2000
UBC9	Abways	Rabbit	CY5571	1:2000
Ubiquitin	Abclonal	Rabbit	A19686	1:2000
AARS1	Proteintech	Rabbit	17394-1-AP	1:2000
ACSL4	Abcam	Rabbit	ab155282	1:20000
GAPDH (HRP Conjugated)	Servicebio	/	ZB15004-HRP-100	1:5000
Anti-L-Lactyl Lysine	PTM Bio	Rabbit	PTM-1401RM	1:1000
Anti-D-Lactyl Lysine	PTM Bio	Rabbit	PTM-1429RM	1:1000
SUMO2-K11la	PTM Bio	Rabbit	Customized	1:1000

CRISPR-Cas9 mediated SUMO2-K11R mutation knock-in

Target	Sequence	Producer
siKu70-1	UUCAGGUGACUCCUCCAGGTT	RIBOBIO
siKu70-2	UUCUCUUGGUAACUUUCCCTT	RIBOBIO
siXRCC4-1	AUAUGUUGGUGAACUGAGATT	RIBOBIO
siXRCC4-2	UCUUGGGACAGAACCUAAATT	RIBOBIO
siLig4-1	CGACCUUUUAGACUCAAUUTT	RIBOBIO
siLig4-2	GCUAGAUGGUGAACGUAUGTT	RIBOBIO
sgRNA	CCTATTGTGAACGACAGGTG	Sangon Biotech
Repair template	GATTTTTGAAGCAACTAGGGCTGCTGCTGCTTTATAAAAC GTGTTCGTTGATCACATTTCCCAAATTATACTTTTATTTTTC TTTTAACAGGAAGGAGTCAGGACTGAGAACAACGATCAT ATTAATTTGAAGGTGGCGGGGCAGGATGGTTCTGTGGTGC AGTTTAAGATTAAGAGGCATACACCACTTAGTAAACTAAT GAAAGCCTACTGTGAGAGACAAGTGAGGAATTCATATGTG TACTTCCACTGAATAAAACTATTTTTAAAAATCTTATTTTCA TGGGAACAAAAACTAGACTAGCACATTACAGTGACTTTTT GATGGGATGAATATGCACTTGGCACGTCAGTTTTGTCGTG CACTTGGTATATTGATAGCTCGTGAAAACCCTTAGGATGAT TTGGTTAGTATTATATGTGGTTCCAGTCCTGTGCCA	Sangon Biotech

Primary antibodies for IHC	Producer	Source	ID/ Number	Dilution
4-HNE	Abcam	Mouse	ab48506	1:50
SUMO2-K11la	PTM Bio	Rabbit	Customized	1:500

Primary antibodies for IF	Producer	Source	ID/ Number	Dilution
SUMO2	Abways	Rabbit	AY9566	1:200
ACSL4	Proteintech	Mouse	66617-1-Ig	1:200

CRISPR-Cas9 mediated ACSL4-K500R mutation knock-in

Target	Sequence	Producer
siKu70-1	UUCAGGUGACUCCUCCAGGTT	RIBOBIO
siKu70-2	UUCUCUUGGUAACUUUCCCTT	RIBOBIO
siXRCC4-1	AUAUGUUGGUGAACUGAGATT	RIBOBIO
siXRCC4-2	UCUUGGGACAGAACCUAAATT	RIBOBIO
siLig4-1	CGACCUUUUAGACUCAAUUTT	RIBOBIO
siLig4-2	GCUAGAUGGUGAACGUAUGTT	RIBOBIO
sgRNA	TAGTATAGTCAGTTACTGTG	Sangon Biotech
Repair template	CAATGGAATCCATTGCACTATTGAAGTGGAGTTGAAATAC TGAAAAATGATCACACTTTGCACTCCTTCTTTTGTCATTTT TCTGACTCTGGTTCTAACTCTAATATTCCCAGTGCATTGGT ACAAATCTGGAATGTGGTATGCTAATATCTCTGTTACTTTTT CCCTCACCGTCACTGACTACACTACTGGCAGAGTTGGAGC ACCTCTTATTTGCTGTGAAATTAAGCTAAGAGACTGGCAA GAAGGTAAGAATTTTATGAAAAGCTCTAGTGATTCAAGAT ATGCTGAAAACTACTGTCAGCTTTATGACTTTCATTTCTTT AAAGTCTGGGGAAGCAAATAATTCTCTGCTGTTTTGCTGT TGCCTGTCCCC	Sangon Biotech

SiRNA	Target sequence	Producer
Human si-SUMO2 #1	GCATACACCACTTAGTA	RIBOBIO
Human si-SUMO2 #2	GGAGGATGAAGATACAA	RIBOBIO
Human si-HDAC1 #1	GCGACTGTTTGAGAACC	RIBOBIO
Human si-HDAC1 #2	GGGATCGGTTAGGTTGC	RIBOBIO
Human si-AARS1 #1	GCAGTGAGATCCACTACGA	RIBOBIO
Human si-AARS1 #2	GTTTGGCATTCCCATTGAA	RIBOBIO
Human si-EP300	CAATTCCGAGACATCTGAGA	RIBOBIO

Human si-CBP	CCCGATAACTTTGTGATGT	RIBOBIO
Human si-AARS2	CCATCATACCTTCTTTGAAAT	RIBOBIO
Human si-KAT2A	GCTACCTACAAGGTCAATT	RIBOBIO
Human si-KAT2B	CCTGTGGTTGAAGGCTCTTTGGAAA	RIBOBIO
Human si-KAT5	TCGAATTGTTTGGGCACTGAT	RIBOBIO
Human si-KAT8	AGCTTTTCCTGGACCATAAGACA	RIBOBIO
Human si-HDAC2	GCATCAGGATTCTGTTACG	RIBOBIO
Human si-HDAC3	CCAAGAGTCTTAATGCCTT	RIBOBIO
Human si-SIRT1	TTCTGAAATATTCAATATCAA	RIBOBIO
Human si-SIRT2	GAGGCCATCTTTGAGATCAGCTATT	RIBOBIO
Human si-SIRT3	TAGGAAAACTGTTGAATTCTAAA	RIBOBIO

**Primers for qRT-PCR**	**Sequence**	**Producer**
ACTINB	F: 5’- CTGGGACGACATGGAGAAAA -3’	Sangon Biotech
	R: 5’- AAGGAAGGCTGGAAGAGTGC -3’	
SUMO2	F: 5’- CTTGTGTGCTCGTTTGGTGC -3’	Sangon Biotech
	R: 5’- GTTGCCCGTCAAATCGGAAT -3’	
AARS1	F: 5’- AGGTGGTGGCAAGGATGTGTCT -3’	Sangon Biotech
	R: 5’- GCTCTTGGCTGGACGGATGGAT -3’	
HDAC1	F: 5’- CGCTCCATCCGTCCAGATAACA -3’	Sangon Biotech
	R: 5’- GCCACAGAACCACCAGTAGACA -3’	
EP300	F: 5’- TTCAGCCAAGCGGCCTAAA -3’	Sangon Biotech
	R: 5’- CGCCACCATTGGTTAGTCCC -3’	
CBP	F: 5’- CAACCCCAAAAGAGCCAAACT -3’	Sangon Biotech
	R: 5’- CCTCGTAGAAGCTCCGACAGT -3’	
AARS2	F: 5’- GTTCAAGCCAATCTTTCTGGGC -3’	Sangon Biotech
	R: 5’- CAGGTCGTTATGGTGTCCTCC -3’	
KAT2A	F: 5’- CTCTGCCTTAACTACTGGAAGC -3’	Sangon Biotech
	R: 5’- GCCATCTGGTGTAATTGACCTTG -3’	
KAT2B	F: 5’- CGGATCGCCGTGAAGAAGG -3’	Sangon Biotech
	R: 5’- CATTGCATTTACAGGACTCCTCT -3’	
KAT5	F: 5’- GGGGAGATAATCGAGGGCTG -3’	Sangon Biotech
	R: 5’- TCCAGACGTTTGTTGAAGTCAAT -3’	
KAT8	F: 5’- CAGCAGAAGTGATCCAGTCTCG -3’	Sangon Biotech
	R: 5’- TTGGTCAGTGCGAGTCGGTTCT -3’	
HDAC2	F: 5’- GGAGGAGGCTACACAATCCG -3’	Sangon Biotech
	R: 5’- TCTGGAGTGTTCTGGTTTGTCA -3’	
HDAC3	F: 5’- GCCAAGACCGTGGCGTATT -3’	Sangon Biotech
	R: 5’- GTCCAGCTCCATAGTGGAAGT -3’	
SIRT1	F: 5’- TAGCCTTGTCAGATAAGGAAGGA -3’	Sangon Biotech
	R: 5’- ACAGCTTCACAGTCAACTTTGT -3’	

SIRT2	F: 5’- TGCGGAACTTATTCTCCCAGA -3’	Sangon Biotech
	R: 5’- GAGAGCGAAAGTCGGGGAT -3’	
SIRT3	F: 5’- ATCCCGGACTTCAGATCCCC -3’	Sangon Biotech
	R: 5’- CAACATGAAAAAGGGCTTGGG -3’	

**Primary antibodies for multiplex-IHC (mIHC)**	**Producer**	**Source**	**ID/ Number**	**Dilution**
CD8	ServiceBio	Rabbit	GB115692	1:400
CD4	ServiceBio	Rabbit	GB15064	1:200
SUMO2-K11la	PTM Bio	Rabbit	Customized	1:500
